# EffAtt-LungNet: An attention-guided EfficientNet framework for three-class lung histopathology classification

**DOI:** 10.1371/journal.pone.0359302

**Published:** 2026-10-05

**Authors:** Muhammad Ahsan Jamil, Malik Daler Ali Awan, Fatima Bukhari, Malik Muhammad Saad Missen, Nadeem Iqbal Kajla

**Affiliations:** 1 Department of Software Engineering, Faculty of Computing, The Islamia University of Bahawalpur, Bahawalpur, Pakistan; 2 Department of Computer Science, National University of Modern Languages (NUML), Islamabad, Pakistan; 3 School of Informatics and Creative Arts, Dundalk Institute of Technology, Dundalk, Ireland; West China Hospital of Sichuan University, CHINA

## Abstract

This study presents EffAtt-LungNet, an attention-guided deep learning framework for three-class lung histopathology classification. The model combines an EfficientNetB3 backbone with the Convolutional Block Attention Module (CBAM) to refine features and improve discrimination among benign lung tissue, lung adenocarcinoma, and lung squamous cell carcinoma. Experiments used the publicly released LC25000 lung subset, which contains 15,000 images across three classes and was itself produced by augmenting a smaller collection of underlying histopathology images. We generated 10,500 additional fixed variants, creating a balanced working corpus of 25,500 images with training, validation, and test partitions of 15,300, 2,550, and 7,650 images. EffAtt-LungNet reached 99.86% accuracy, 99.86% precision, 99.86% recall, 99.86% F1 score, and 0.9997 AUC on the image-level test partition. Performance was stable across five random seeds (mean accuracy 99.86±0.02%), and paired errors differed from those of the EfficientNetB3 backbone (McNemar’s test, $\chi^{2}=47.27$, *p* < 0.001). Because pre-augmentation source identifiers for the released LC25000 images were unavailable, independence at the earliest source-image level could not be confirmed. The results therefore represent internal tile-level benchmark performance and require validation on independent, non-augmented, multi-centre datasets before any translational or clinical claim can be made.

## Introduction

Lung cancer is one of the leading causes of cancer mortality worldwide, accounting for approximately 1.8 million deaths each year and close to 25% of all cancer deaths [1]. Most cases are non-small cell lung cancer (NSCLC), which represents about 85% of diagnoses and includes adenocarcinoma, squamous cell carcinoma, and large cell carcinoma [2]. Subtype identification affects diagnosis, prognosis, and treatment planning. Histopathological examination remains central to lung cancer confirmation and subtyping, but interpretation is difficult because of complex tissue architecture and subtle cellular variation in microscopic images [3, 4].

The conventional workflow relies on expert pathologists to separate benign from malignant patterns and, where relevant, assign a histological subtype. This process is time-intensive and subject to inter-observer variability when morphological differences are small [5,6]. Growing volumes of digital pathology data motivate computational tools that can support image interpretation consistently and efficiently.

Deep learning has become effective for medical image analysis by learning discriminative features directly from image data [7,8]. Convolutional neural networks (CNNs) have been applied widely to histopathological classification and have produced strong results in lung cancer analysis [9,10]. Open problems remain, including limited interpretability, sensitivity to visually irrelevant regions, and inconsistent performance in multi-class settings [11,12]. The LC25000 dataset contains five balanced classes (two colon and three lung categories). This study retained only the three lung categories, benign lung tissue, lung adenocarcinoma, and lung squamous cell carcinoma, to build an LC25000-derived augmented lung corpus. The aim was to test whether attention-guided feature refinement improves tile-level discrimination among these three categories under a controlled benchmark. The study does not claim clinical deployment readiness; it reports a controlled evaluation of the proposed model on an augmented histopathology corpus.

EffAtt-LungNet combines an EfficientNetB3 backbone with CBAM to examine whether attention-guided refinement improves three-class classification of benign tissue, adenocarcinoma, and squamous cell carcinoma while keeping computational cost low. Grad-CAM is used for qualitative inspection of predictions. The contribution is an evaluation of whether attention improves tile-level lung histopathology classification under a defined experimental protocol, not a claim of clinical readiness.

### Related work

Artificial intelligence has been applied increasingly to lung cancer detection and classification, across radiological and histopathological imaging, using methods that range from conventional machine learning to deep neural networks. Because this study addresses histopathological lung image classification, the most relevant prior work is in that setting, while computed tomography (CT)-based studies provide background rather than direct benchmarks.

Early work often used handcrafted features with conventional classifiers. Rehman et al. [12] combined Local Binary Pattern and Discrete Cosine Transform features with Support Vector Machines and k-Nearest Neighbors, reporting 93% and 91% accuracy on CT datasets [13,14]. Engineered texture features captured useful information, but performance depended on manual feature design and task-specific preprocessing.

Deep learning reduced this dependence on handcrafted features by learning features from image data directly [15,16]. CNNs support end-to-end learning and model spatial patterns at multiple levels of abstraction. Poornima and Anand [17] used a CNN with Gaussian blur preprocessing and augmentation for subtype classification, reporting 97.74% accuracy, 98.96% precision, and 0.98 AUC. Sherin et al. [18] reported that carefully designed pipelines can improve deep learning based screening. Hasanuzzaman [4] proposed an edge-enhanced CNN and reported an increase from 85% to 91% accuracy relative to grayscale inputs, indicating that preprocessing affects downstream classification, consistent with related residual-network work on CT images [19].

Several CNN-based and transfer-learning methods have reported strong histopathological results. Rajaram et al. [20,21] applied VGG-16 to histopathological lung images and reported 97.5% training and 98.5% validation accuracy. Architectures such as VGG-16 are computationally heavy and less attractive when efficiency is a design goal [22]. Kanwal et al. [3,23] used a ResNet18-enhanced CNN for lung tissue classification and reported 99% accuracy. Keerthi et al. [24] compared several pre-trained models and reported peak accuracy of 97% with DenseNet.

Attention mechanisms have been used to focus feature selection on diagnostically relevant regions. Illakiya et al. [8] combined DenseNet169 with CBAM on 15,000 histopathological images and reported 99.7% accuracy. This indicates that attention modules are useful in histopathology, where discriminative patterns are often localized and visually subtle.

Transformer-based models have also been studied. Imran et al. [2] proposed a hybrid CNN-transformer model for NSCLC classification on LC25000 and reported 98.8% accuracy, 0.980 F1-score, and 0.991 specificity. Sallagundla et al. [25] fine-tuned Vision Transformers for lung and colon histopathology and reported 100% accuracy on their test sets. These results depend on the specific datasets, class definitions, and evaluation settings used.

EfficientNet models are attractive because compound scaling balances model size and accuracy. Kumar and Nelson [10] used EfficientNetB3 for histopathological classification and reported 99.93% accuracy. Patharia and Sethy [1] combined EfficientNetB0 with SVM for carcinoma grading. These studies show that EfficientNet variants are suitable for lung image analysis when efficiency matters.

Interpretability has become important in medical AI. Sumithra et al. [7] added Grad-CAM visual interpretation to a CNN for chest disease classification. Conventional machine learning pipelines remain competitive baselines for lung cancer prediction [26], and dedicated NSCLC classifiers based on deep networks have also been examined [27]. Beyond histopathology, Wehbe et al. [5] presented a YOLOv8-based detection and TNM staging framework and reported 97.1% mean Average Precision and 98% stage classification accuracy. CT-based detection and staging address a different task from histopathological subtype classification and are treated here as context rather than baselines.

Strong performance has been reported across CNNs, attention-augmented models, transformers, and EfficientNet variants. Direct comparison across studies is difficult because reported results use different datasets, image modalities, class definitions, and evaluation protocols. For a histopathology-focused study, the most relevant prior work is the subset of methods evaluated on histopathological lung tissue, especially those using LC25000 or comparable three-class settings. The objective of this study is to test whether an EfficientNetB3 backbone combined with CBAM improves three-class lung histopathology classification while preserving efficiency, using an expanded corpus derived from the publicly released LC25000 lung subset.

## Materials and methods

This section describes the framework used to develop and evaluate EffAtt-LungNet for lung histopathology classification. The pipeline covers dataset preparation, preprocessing, network design, optimization, interpretability analysis, and evaluation. The workflow was designed for reproducibility and a transparent basis for assessment, and is shown in Fig 1.

**Fig 1:**
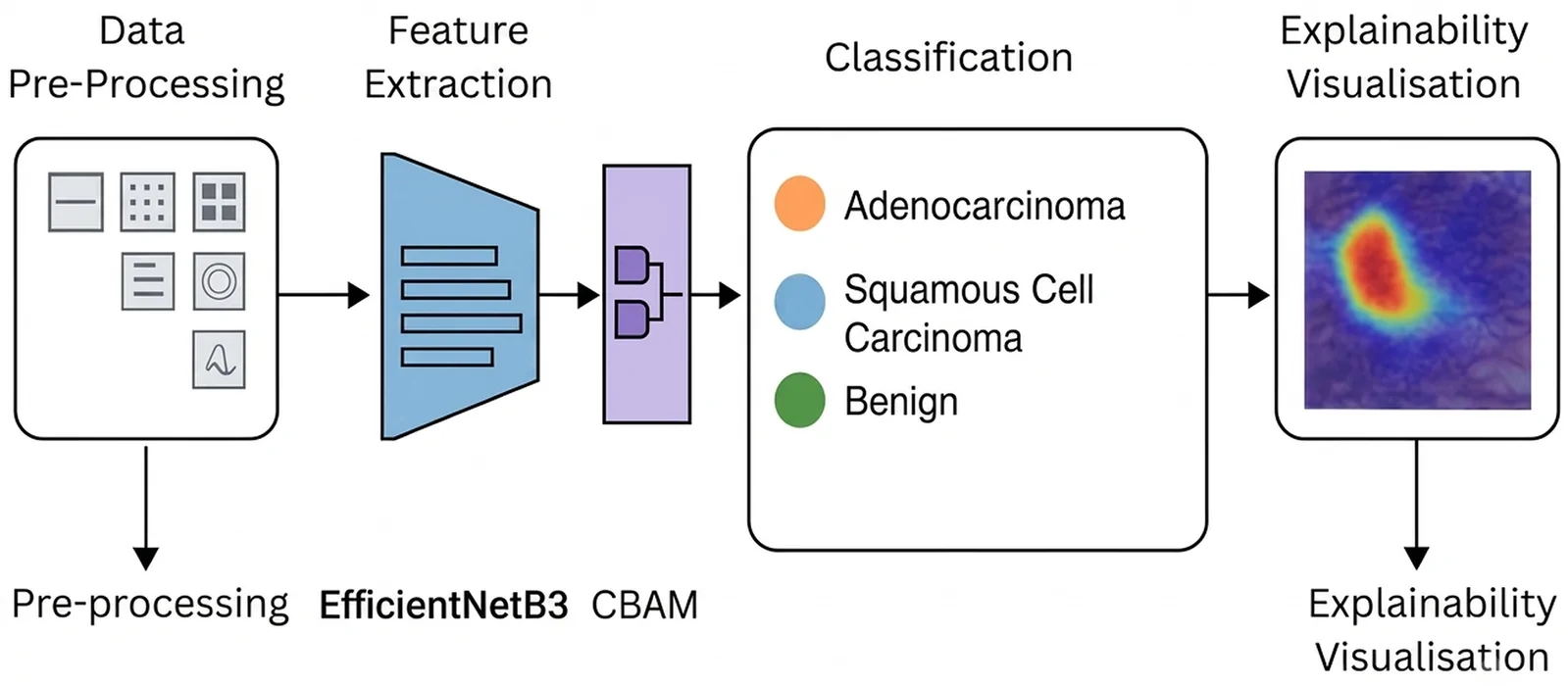
Overall workflow of the proposed EffAtt-LungNet framework.

### Dataset description and splitting protocol

Experiments used the three lung categories in the publicly released LC25000 dataset: benign lung tissue, lung adenocarcinoma, and lung squamous cell carcinoma [28]. The released lung subset contains 15,000 images, with 5,000 images per class at a spatial resolution of 768×768 pixels. These images represent the publicly released LC25000 dataset, which was constructed by cropping and augmenting a smaller underlying histopathology image collection. We therefore use the term ‘released LC25000 images’ rather than ‘original images. For the present study, 3,500 additional fixed, label-preserving variants were generated per lung class from the released LC25000 images. This step increased each class from 5,000–8,500 images and produced a balanced working corpus of 25,500 images.

To prevent train–test leakage from the additional augmentation performed in this study, partition assignment was controlled at the immediate-parent image level. Each publicly released LC25000 image and all fixed variants generated from that image were assigned exclusively to a single training, validation, or test partition. Consequently, no parent image and its additional variants generated in the present study were shared across partitions. The resulting stratified split contained 15,300 training, 2,550 validation, and 7,650 test images

The task was formulated as tile-level three-class classification rather than slide-level or patient-level diagnosis. All reported findings therefore describe internal benchmark performance on an LC25000-derived image corpus, not real-world clinical diagnostic performance.

The three classes are shown in Fig 2, and the split statistics are summarized in Table 1.

**Table 1:** Statistics of the LC25000-derived augmented lung corpus used in this study.

Class	Released LC25000 images	Additional variants	Working corpus	Train	Validation	Test
Benign (LUBEN)	5000	3500	8500	5100	850	2550
Adenocarcinoma (LUAD)	5000	3500	8500	5100	850	2550
Squamous cell carcinoma (LUSC)	5000	3500	8500	5100	850	2550
**Total**	**15000**	**10,500**	**25,500**	**15300**	**2550**	**7650**

The released LC25000 images were themselves created by augmenting a smaller underlying image collection. “Additional variants” refers only to augmentation performed in the present study.

**Fig 2:**
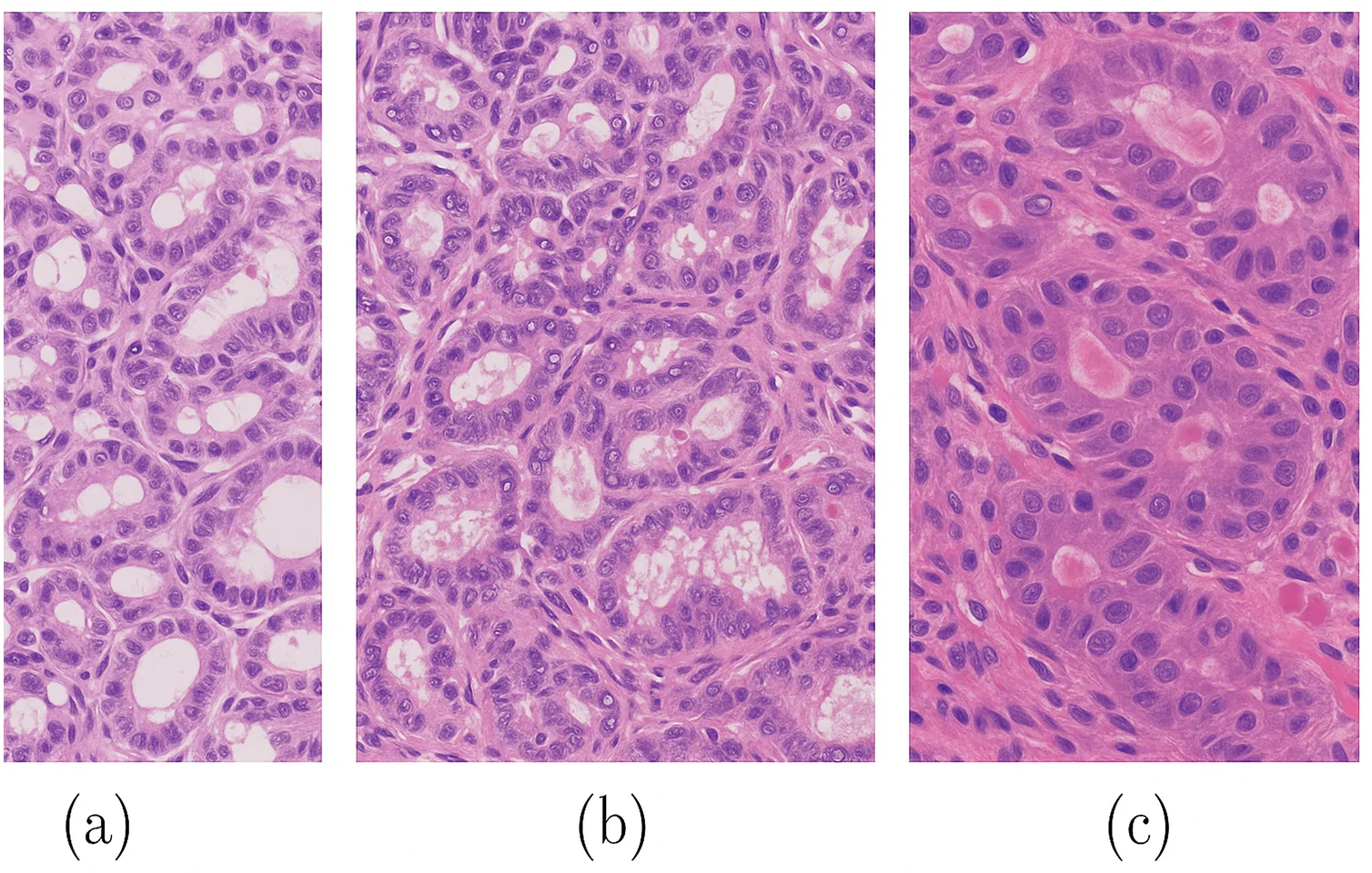
Representative examples from the LC25000 lung histopathology subset. (a) benign lung tissue, (b) lung adenocarcinoma, and (c) lung squamous cell carcinoma. The three classes show distinct morphology that supports automated multi-class discrimination.

### Data preprocessing

Two forms of augmentation performed in this study were kept separate. Offline augmentation built the fixed working corpus by expanding each released LC25000 class from 5,000–8,500 images. Stochastic training-time augmentation was applied only to the training partition after partition assignment. Validation and test images underwent deterministic resizing and normalisation only and were not subjected to random augmentation during evaluation.

All input images were resized before training and inference. Each 768×768 tile was resized, not cropped, to 224×224 pixels using bilinear interpolation; no grid-based sub-tiling was performed. This preserves the full tissue field of view at the resolution expected by the EfficientNetB3 stem and balances computational cost against preservation of tile-level morphology. The resizing operation is defined in Eq (1):


$$\begin{array}{c} {𝐗}_{r}=Resize\;\left(𝐗,224,224\right), \end{array}$$ (1)

where 𝐗 is the original image tile and ${𝐗}_{r}$ is the resized image.

After resizing, channel-wise normalization was applied using the mean and standard deviation from ImageNet pretrained initialization, as defined in Eq (2):


$$\begin{array}{c} {𝐗}_{n}=\frac{{𝐗}_{r}-\mu }{\sigma }, \end{array}$$ (2)

where μ=[0.485,0.456,0.406] and σ=[0.229,0.224,0.225].

After the fixed corpus was built and the split completed, stochastic augmentation was applied only to the training subset to improve generalization. Validation and test subsets used deterministic resizing and normalization only. The augmentation is expressed in Eq (3):


$$\begin{array}{c} {𝐗}_{a}=T({𝐗}_{n}),\;T\sim 𝒯, \end{array}$$ (3)

where 𝒯 is the training-time augmentation distribution. The policy increased appearance variability while preserving tissue structure.

The transformations in Table 2 define the offline augmentation performed in this study; they were applied once to the released 768×768 LC25000 images before the split. This stage is separate from the stochastic training-time augmentation of Eq (3), which is applied only to the training partition. Offline augmentation creates a fixed expanded corpus used by all models, whereas training-time augmentation generates a new perturbation during each training pass. Neither step resolves possible relationships among derivatives created during the original construction of LC25000.

**Table 2:** Offline label-preserving augmentation used to expand each lung class from 5,000 to 8,500 images. Operations were applied to the released 768×768 LC25000 images before the train/validation/test split.

Operation	Setting
Random rotation	$\left\{90^{\circ },180^{\circ },270^{\circ }\right\}$
Horizontal / vertical flip	applied
Stain / colour jitter	± 20%
Fixed variants per released image	0.7 on average (3,500 new tiles per class)
Output resolution	768×768

### EffAtt-LungNet architecture

EffAtt-LungNet combines an EfficientNetB3 backbone with convolutional block attention modules and a lightweight classification head. The design targets improved representation learning for lung histopathology at low computational cost. The architecture is shown in Fig 3.

**Fig 3:**
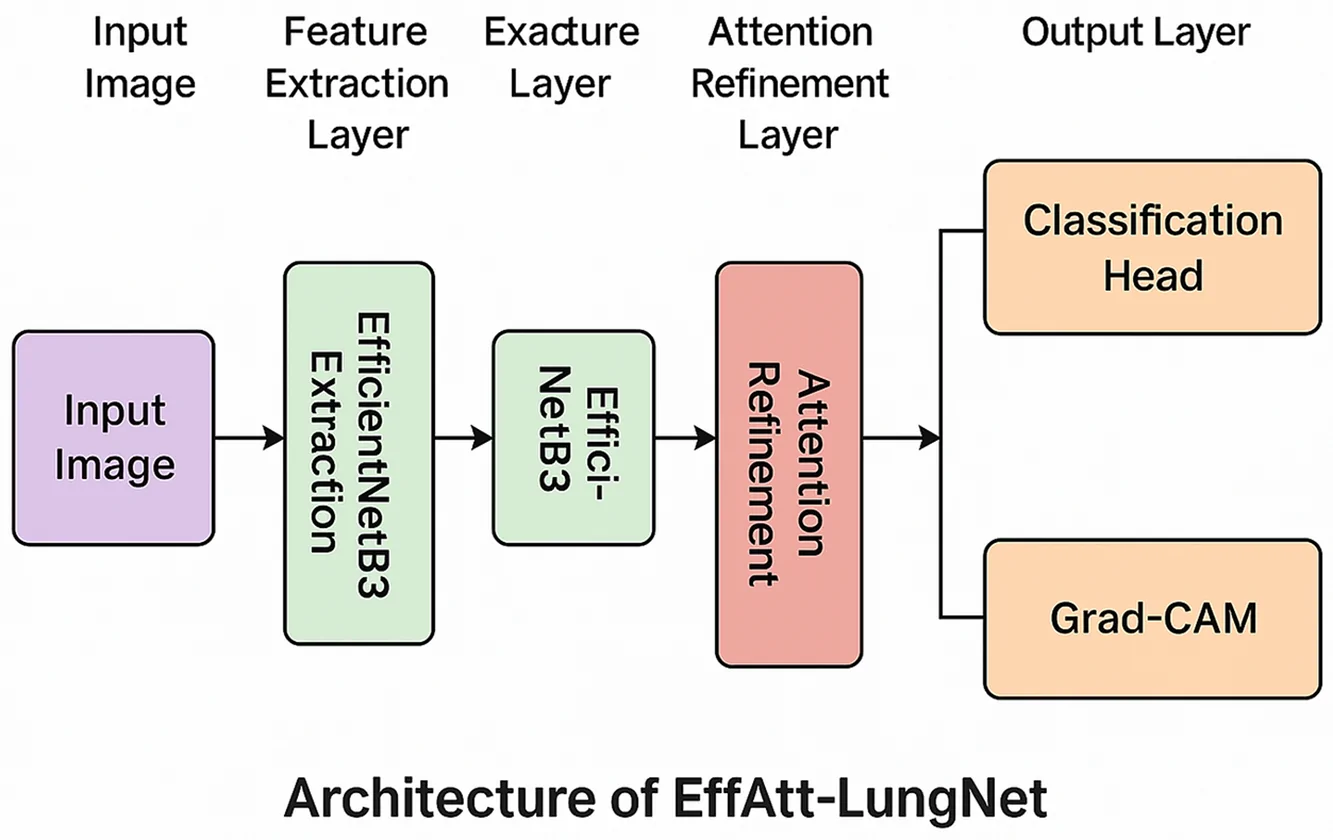
Architecture of the proposed EffAtt-LungNet framework. EfficientNetB3 extracts hierarchical features, CBAM enhances informative responses through channel and spatial attention, and the classification head produces class probability estimates.

CBAM modules were inserted after EfficientNetB3 stages 3 and 5. This placement was selected from validation performance and the ablation reported below.

### EfficientNetB3 backbone

EfficientNetB3 was used as the feature extractor because it balances representational capacity and computational cost. The EfficientNet family jointly scales depth, width, and input resolution according to Eq (4):


$$\begin{array}{c} d=\alpha^{\phi },\;w=\beta^{\phi },\;r=\gamma^{\phi }, \end{array}$$ (4)

subject to the compound scaling constraint in Eq (5):


$$\begin{array}{c} \alpha \beta^{2}\gamma^{2}\approx 2. \end{array}$$ (5)

For an augmented input image ${𝐗}_{a}$, the backbone feature extractor is defined in Eq (6):


$$\begin{array}{c} 𝐅=f_{b3}({𝐗}_{a};\theta_{b3}), \end{array}$$ (6)

where $𝐅\in {\mathbb{R}}^{H^{\prime }\times W^{\prime }\times C^{\prime }}$ is the extracted feature tensor and $\theta_{b3}$ are the trainable backbone parameters.

### CBAM-based attention refinement

CBAM models channel attention followed by spatial attention. For a feature tensor 𝐅, the channel attention map is computed by Eq (7):


$$\begin{array}{c} {𝐌}_{c}=\sigma \;\left(MLP(GAP(𝐅))+MLP(GMP(𝐅))\right), \end{array}$$ (7)

and the channel-refined tensor is obtained through Eq (8):


$$\begin{array}{c} {𝐅}_{c}={𝐌}_{c}⊗𝐅, \end{array}$$ (8)

where σ(·) is the sigmoid activation and ⊗ is element-wise multiplication. In practical terms, channel attention assigns larger weights to feature channels that contribute more strongly to the current representation and smaller weights to less responsive channels.

Spatial attention is applied to the channel-refined representation. The spatial attention map is defined by Eq (9):


$$\begin{array}{c} {𝐌}_{s}=\sigma \;\left(f^{7\times 7}\;\left[{AvgPool}_{c}({𝐅}_{c});{MaxPool}_{c}({𝐅}_{c})\right]\right), \end{array}$$ (9)

and the final attention-refined representation is obtained from Eq (10):


$$\begin{array}{c} {𝐅}_{att}={𝐌}_{s}⊗{𝐅}_{c}. \end{array}$$ (10)

Spatial attention estimates which locations within the channel-refined feature map receive greater weight. Channel attention therefore refines which feature responses are retained, and spatial attention refines where those responses are weighted most strongly. These weights are internal model responses and are not pathologist-validated localizations.

### Classification head

The attention-refined feature tensor ${𝐅}_{att}$ is converted into a compact descriptor by global average pooling, as in Eq (11):


$$\begin{array}{c} 𝐳=GAP({𝐅}_{att}). \end{array}$$ (11)

The pooled representation is processed by a lightweight fully connected head. The first hidden representation is computed by Eq (12):


$$\begin{array}{c} {𝐡}_{1}=ReLU({𝐖}_{1}𝐳+{𝐛}_{1}), \end{array}$$ (12)

the second by Eq (13):


$$\begin{array}{c} {𝐡}_{2}=ReLU({𝐖}_{2}{𝐡}_{1}+{𝐛}_{2}), \end{array}$$ (13)

and the class probability distribution by the softmax in Eq (14):


$$\begin{array}{c} \hat{𝐲}=softmax({𝐖}_{3}{𝐡}_{2}+{𝐛}_{3}), \end{array}$$ (14)

where $\hat{𝐲}\in {\mathbb{R}}^{3}$ is the predicted distribution over the three classes.

### Training objective

The model was optimized with a composite objective that combines cross-entropy loss, focal loss, and $\ell_{2}$ regularization, defined in Eq (15):


$$\begin{array}{c} {ℒ}_{total}={ℒ}_{ce}+\lambda_{f}{ℒ}_{focal}+\lambda_{r}\|\theta {\|}_{2}^{2}, \end{array}$$ (15)

where the cross-entropy term is given by Eq (16):


$$\begin{array}{c} {ℒ}_{ce}=-\frac{1}{N}\sum_{i=1}^{N}\sum_{j=1}^{3}y_{ij}\log({\hat{y}}_{ij}), \end{array}$$ (16)

and the focal term by Eq (17):


$$\begin{array}{c} {ℒ}_{focal}=-\frac{1}{N}\sum_{i=1}^{N}\sum_{j=1}^{3}\alpha_{j}(1-{\hat{y}}_{ij}{)}^{\gamma }y_{ij}\log({\hat{y}}_{ij}). \end{array}$$ (17)

The focusing parameter was fixed at γ=2, while $\lambda_{f}$ and $\lambda_{r}$ were selected using validation macro F1. Because the classes were numerically balanced, the focal component was included to reduce the relative contribution of well-classified examples and give greater weight to difficult examples rather than to correct class imbalance. The same selected objective was used in all five random-seed runs. The archived manuscript files did not retain the numerical values or validation-search ranges for $\lambda_{f}$ and $\lambda_{r}$ or the class coefficients $\alpha_{j}$; this limits exact reproduction of the composite objective from the manuscript alone.

### Training protocol and optimization

Training used the AdamW optimizer with cosine annealing learning-rate scheduling, warmup, gradient clipping, and early stopping. The checkpoint with the highest validation macro F1 score was retained for final evaluation on the isolated test set. The main settings are summarized in Table 3.

**Table 3:** Training configuration used for model optimization.

Parameter	Value
Batch size	32
Initial learning rate	$3\times 10^{-4}$
Optimizer	AdamW
AdamW coefficients	$\beta_{1}=0.9,\;\beta_{2}=0.999$
Weight decay	$1\times 10^{-4}$
Maximum epochs	200
Learning rate schedule	Cosine annealing
Warmup epochs	10
Early stopping	Patience = 20
Gradient clipping	1.0

The cosine annealing schedule is defined by Eq (18):


$$\begin{array}{c} \eta_{t}=\eta_{\min}+\frac{1}{2}\left(\eta_{\max}-\eta_{\min}\right)\left(1+\cos\left(\frac{t}{T}\pi \right)\right), \end{array}$$ (18)

where $\eta_{\max}$ is the initial learning rate, $\eta_{\min}$ the minimum learning rate, *t* the current epoch, and *T* the total number of scheduled epochs. The end-to-end optimiza*t*ion and model selection procedure is given in Algorithm 1. All models, including the proposed model and reimplemented baselines, were trained and evaluated using the same corpus, immediate-parent grouping procedure, and preprocessing protocol.

### Model interpretability

Grad-CAM was applied to the final convolutional layer before global average pooling. For class *c*, the Grad-CAM weights are computed by Eq (19):


$$\begin{array}{c} \alpha_{k}^{c}=\frac{1}{Z}\sum_{i}\sum_{j}\frac{\partial S^{c}}{\partial A_{ij}^{k}}, \end{array}$$ (19)

where $S^{c}$ is the pre-softmax score for class *c*, $A^{k}$ is the *k*th activation map, and *Z* is the spatial normalization factor. The localization map is obtained from Eq (20):


$$\begin{array}{c} L_{GradCAM}^{c}=ReLU\left(\sum_{k}\alpha_{k}^{c}A^{k}\right). \end{array}$$ (20)

Heatmaps were min–max normalised and resized to the input resolution for visualization. CBAM channel and spatial attention maps were also examined after normalization. These visualizations were used only as illustrative, qualitative examples of model behaviour. They were not compared with pathologist annotations or evaluated using localization metrics and were not treated as evidence of diagnostic localization.

### Evaluation metrics and validation protocol

Model development followed a three-stage protocol. First, hyperparameters were optimized using the training and validation partitions. Second, the checkpoint with the highest validation macro F1 score was selected. Third, the selected checkpoint was evaluated once on the image-level test partition of 7,650 images. This partition included 2,550 fixed images per class and was not used during training or model selection. Additional variants generated in the present study were kept in the same partition as their immediate parent LC25000 image. No separate exact-hash or perceptual-similarity audit was part of the original experimental protocol. Because pre-augmentation source identifiers for the released LC25000 images were unavailable, this procedure does not exclude related derivatives from the original LC25000 augmentation process appearing in different partitions.

Performance was measured using accuracy, macro precision, macro recall, macro F1 score, one-versus-rest area under the receiver operating characteristic curve, and Matthews correlation coefficient (MCC). Because the final split was class-balanced, macro-averaged metrics were emphasised alongside overall accuracy. A confusion matrix was reported in absolute counts to show correct and incorrect predictions across the three classes. All reported test metrics are point estimates from a single image-level split. Multi-seed stability and test-set bootstrap confidence intervals are also reported in the Results.

**Algorithm 1. Training procedure for EffAtt-LungNet**

**Require:** Training set ${𝒟}_{train}$, validation set ${𝒟}_{val}$, hyperparameters ℋ

**Ensure:** Optimized model parameters $\theta^{*}$

1: Initialize the EfficientNetB3 backbone, CBAM modules, and classification head

2: Initialize the AdamW optimizer with learning rate $\eta_{\max}$

3: Set early stopping patience to *P*

4: best_score←−∞

5: **for** epoch = 1 to *E* **do**

6:   Set the model to training mode

7:   **for** each mini batch $(𝐗,𝐲)\in {𝒟}_{train}$ **do**

8:     ${𝐗}_{a}\leftarrow OnlineAugment(𝐗)$

9:     $𝐅\leftarrow f_{b3}({𝐗}_{a})$

10:     ${𝐅}_{att}\leftarrow CBAM(𝐅)$

11:     $\hat{𝐲}\leftarrow Head({𝐅}_{att})$

12:     Compute ${ℒ}_{total}$ using Eq (15)

13:     Backpropagate the loss

14:     Update model parameters using AdamW

15:   **end for**

16:   Update the learning rate using Eq (18)

17:   Evaluate the model on ${𝒟}_{val}$

18:   **if** validation macro F1 improves **then**

19:     Save the current model parameters

20:     best_score←current_score

21:   **end if**

22:   **if** the early stopping criterion is satisfied **then**

23:     **break**

24:   **end if**

25: **end for**

26: Load the best saved model parameters $\theta^{*}$

27: **return** $\theta^{*}$

## Results

This section reports the experimental findings for EffAtt-LungNet on the three-class lung histopathology task. The analysis covers overall benchmark performance, class-wise behaviour and error structure, discriminative ability, computational efficiency, and the contribution of the proposed components. All results refer to the tile-level benchmark setting used in this study and are not evidence of clinical deployment.

### Experimental setup and reproducibility

All experiments were implemented in PyTorch and executed using the hardware in Table 4. Final test results were computed from the single checkpoint selected on the validation partition by highest validation macro F1 score. All baselines were trained using the same working corpus, immediate-parent grouping procedure, preprocessing pipeline, model-selection criterion, and evaluation protocol. The immediate-parent grouping applies only to variants generated in this study and should not be interpreted as a verified mapping to the earliest images used to create LC25000.

**Table 4:** Experimental environment configuration.

Component	Specification
GPU	NVIDIA A100 (40 GB VRAM) × 4
CPU	Intel Xeon Gold 6248R (3.0 GHz)
RAM	256 GB DDR4–3200
Storage	2 TB NVMe SSD
Framework	PyTorch 1.12.0
CUDA	11.6
Python	3.9.7
Operating system	Ubuntu 20.04.4 LTS

### Primary benchmark performance

EffAtt-LungNet achieved the strongest overall performance among the reimplemented baselines evaluated under the same protocol, with only a limited increase in computational cost. The held-out augmented test partition contained 7,650 fixed tiles, with 2,550 per class.The performance comparison with the reimplemented baseline architectures is summarised in Table 5.

**Table 5:** Performance comparison of the proposed model with baseline architectures.

Architecture	Accuracy (%)	Macro F1 (%)	AUC	MCC
VGG16	96.23	96.37	0.985	0.943
ResNet50	98.34	98.36	0.994	0.975
DenseNet169	98.41	98.42	0.995	0.976
EfficientNetB3	98.97	98.98	0.997	0.985
ViT-B/16	98.64	98.66	0.996	0.980
Swin Transformer	98.73	98.74	0.996	0.981
ConvNeXt	98.82	98.83	0.997	0.982
**EffAtt-LungNet (Ours)**	**99.86**	**99.86**	**0.9997**	**0.998**

Compared with EfficientNetB3, EffAtt-LungNet improved accuracy by 0.89 percentage points and increased MCC from 0.985 to 0.998. Paired classification outcomes differed according to McNemar’s test with continuity correction ($\chi^{2}=47.27$, *p* < 0.001). The absolute accuracy gain was 0.89 percentage points and should be interpreted as a modest benchmark improvement because the EfficientNetB3 baseline was already near the performance ceiling. Although McNemar’s test indicates a difference in paired classification errors, statistical significance in this setting does not imply a large practical or clinical effect. The bootstrap intervals reported in Table 7 quantify uncertainty in the individual performance estimates and should not be interpreted as confidence intervals for the paired model difference. The confusion matrix in Fig 4 shows that EffAtt-LungNet correctly classified 7,639 of 7,650 test images, corresponding to an accuracy of 99.86%. These results remain subject to the unresolved dependence among derivatives in the released LC25000 dataset.

**Fig 4:**
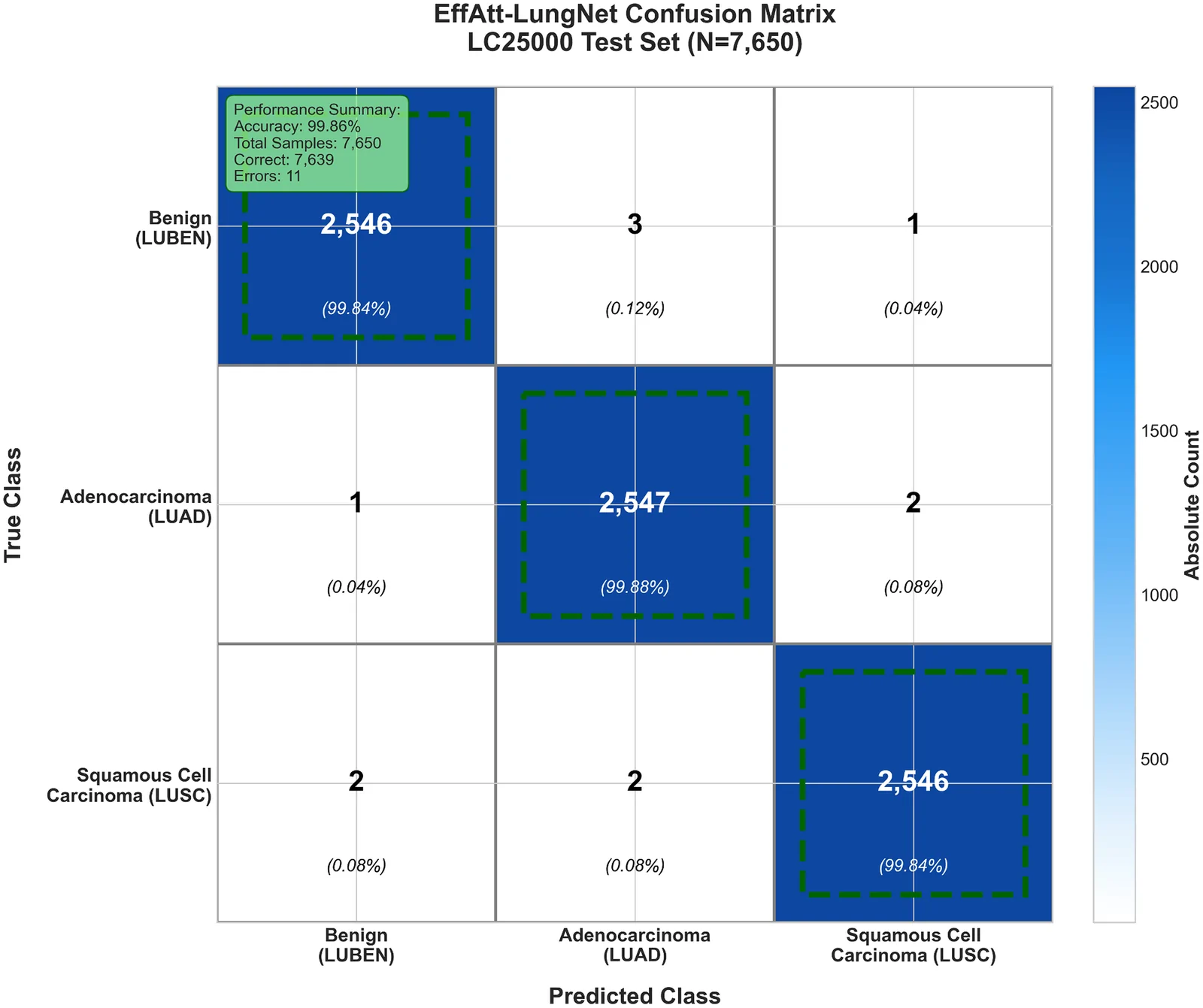
Confusion matrix of EffAtt-LungNet on the held-out LC25000-derived augmented test partition. Counts are reported in absolute numbers.

The error distribution in Table 6 shows that four of the 11 misclassifications occurred between LUAD and LUSC, whereas only three malignant tiles were assigned to the benign class. This is consistent with the pathology, since adenocarcinoma and squamous cell carcinoma can share visual cues at the tile level, especially when glandular differentiation, stromal context, keratinisation, or nuclear atypia are only partially represented within a limited field of view. The small number of benign-versus-malignant errors is relevant because such errors are generally more consequential in downstream diagnostic settings.

**Table 6:** Error analysis on the LC25000-derived augmented test partition.

True class	Pred. Benign	Pred. LUAD	Pred. LUSC	Errors	Error rate (%)
Benign (LUBEN)	2546	3	1	4	0.16
Adenocarcinoma (LUAD)	1	2547	2	3	0.12
Squamous cell carcinoma (LUSC)	2	2	2546	4	0.16
**Total**	**2549**	**2552**	**2549**	**11**	**0.14**

To characterise run-to-run variability, the model was retrained under five random seeds, and the held-out test set was resampled with the non-parametric bootstrap (1,000 resamples). The means, standard deviations, and 95% confidence intervals are summarised in Table 7. The small dispersion across seeds and the narrow bootstrap intervals indicate that the point estimates are stable rather than favourable single-run outcomes.

**Table 7:** Stability of EffAtt-LungNet over five random seeds and test-set bootstrap (1,000 resamples). Values are mean ± standard deviation; brackets give 95% CI.

Metric	Mean ± SD (5 seeds)	95% CI (bootstrap)
Accuracy (%)	99.86 ± 0.02	99.76, 99.93
Macro F1 (%)	99.86 ± 0.02	99.75, 99.93
AUC	0.9997 ± 0.0001	0.9994, 0.9999

As a supplementary analysis, the selected checkpoint was evaluated on 4,500 held-out released LC25000 images, excluding additional variants generated in the present study. Accuracy and macro F1 were both 99.80%. This comparison assesses sensitivity to the extra offline augmentation performed in this study, but it does not represent evaluation on the pre-augmentation images used to construct LC25000 and does not establish independence at that source level.

### Class-wise performance

Per-class precision, recall, F1 score, specificity, and one-versus-rest MCC were computed from the confusion matrix and are shown in Table 8. Performance was balanced across all three categories.

**Table 8:** Class-wise performance on the LC25000-derived augmented test partition.

Class	Precision (%)	Recall (%)	F1 (%)	Specificity (%)	Support	MCC
Benign (LUBEN)	99.88	99.84	99.86	99.94	2550	0.9979
Adenocarcinoma (LUAD)	99.80	99.88	99.84	99.90	2550	0.9976
Squamous cell carcinoma (LUSC)	99.88	99.84	99.86	99.94	2550	0.9979
**Macro average**	**99.86**	**99.86**	**99.86**	**99.93**	**7650**	**0.9978**
**Weighted average**	**99.86**	**99.86**	**99.86**	**99.93**	**7650**	**0.9978**

The close agreement between macro and weighted averages is expected because the evaluation split was class-balanced. The model did not reach high overall accuracy by favouring one category. Precision and recall were uniform across benign tissue, LUAD, and LUSC, which indicates that the decision boundary is well distributed across the three-class structure. The ROC analysis in Table 9 gives AUC values close to 1.0, so the classifier maintained high sensitivity at very low false positive rates across the decision range.

**Table 9:** ROC summary for EffAtt-LungNet on the LC25000-derived augmented test partition.

Setting	AUC	Sensitivity (%)	Specificity (%)
Benign vs. rest	0.9998	99.84	99.94
LUAD vs. rest	0.9997	99.88	99.90
LUSC vs. rest	0.9996	99.84	99.94
**Macro average**	**0.9997**	**99.86**	**99.93**

The multi-seed and bootstrap analysis in Table 7 indicates narrow variability around the point estimates.

### Computational efficiency

A practical benchmark model should combine strong predictive performance with a reasonable computational footprint. Table 10 compares EffAtt-LungNet with representative baselines on parameter count, FLOPs, memory, and inference latency.

**Table 10:** Computational efficiency comparison.

Architecture	Params (M)	FLOPs (G)	Memory (MB)	GPU time (ms)	Accuracy (%)
ResNet18	11.7	1.8	87	18.9	97.85
ResNet50	25.6	4.1	156	31.2	98.34
DenseNet169	14.2	3.4	114	35.7	98.41
EfficientNetB0	5.3	0.4	43	15.8	98.45
EfficientNetB3	12.3	1.8	89	25.4	98.97
ViT-B/16	86.5	17.6	334	54.1	98.64
Swin Transformer	49.6	8.7	187	45.6	98.73
**EffAtt-LungNet**	**13.5**	**2.0**	**96**	**29.5**	**99.86**

EffAtt-LungNet required only 1.2 million additional parameters relative to EfficientNetB3 and increased GPU inference time by 4.1 ms, while improving held-out augmented test accuracy by 0.89 percentage points. Attention refinement therefore improved feature quality without a large computational penalty. The model sits between lightweight convolutional baselines and heavier transformer architectures.

### Ablation study

All ablation experiments used the same corpus and the same partitions as the primary experiment. The ablations isolate the contribution of the attention mechanism and the multi-stage integration strategy, and confirm that the gain did not come from EfficientNetB3 alone but from how attention was introduced into the backbone.

Table 11 shows a monotonic improvement as attention modelling becomes more expressive. The strongest configuration inserts CBAM modules at stages 3 and 5, which indicates that mid-to-late feature refinement is more useful than early attention alone. This is consistent with diagnostically informative lung histomorphology emerging more clearly in semantically richer feature maps than in early, low-level representations.

**Table 11:** Architectural ablation study.

Configuration	Accuracy (%)	Macro F1 (%)	AUC	Params (M)	GPU time (ms)
EfficientNetB3 only	98.97	98.98	0.997	12.3	25.4
+ Channel attention	99.35	99.36	0.998	12.5	26.8
+ Spatial attention	99.47	99.46	0.998	12.6	27.2
+ Single-stage CBAM	99.73	99.74	0.999	12.8	28.1
**+ Multi-stage CBAM (3, 5)**	**99.86**	**99.86**	**0.9997**	**13.5**	**29.5**

The loss ablation in Table 12 shows that focal loss improved optimization beyond plain cross-entropy, particularly for harder or more ambiguous samples. The effect of the objective function was smaller than the effect of the architectural modification, which indicates that the main source of improvement was attention-guided feature refinement rather than the loss design.

**Table 12:** Loss function ablation.

Loss configuration	Accuracy (%)	Macro F1 (%)	Epochs to convergence	Validation loss
Cross-entropy only	99.34	99.35	147	0.0267
Cross-entropy + focal loss	99.67	99.68	126	0.0203
Cross-entropy + label smoothing	99.45	99.46	139	0.0238
Cross-entropy + mixup	99.52	99.53	131	0.0223
**Final objective (CE + focal + $\ell_{2}$)**	**99.86**	**99.86**	**118**	**0.0161**

### Qualitative interpretation

Grad-CAM and CBAM maps were examined as illustrative examples of model behaviour. As shown in Figs 5 and 6, the selected maps contained spatially concentrated responses in visually plausible morphology-associated regions. The maps were not compared with pathologist annotations, and no localization or attribution metric was calculated. They therefore do not establish that highlighted regions correspond to validated diagnostic structures or causal features used by the model.

**Fig 5:**
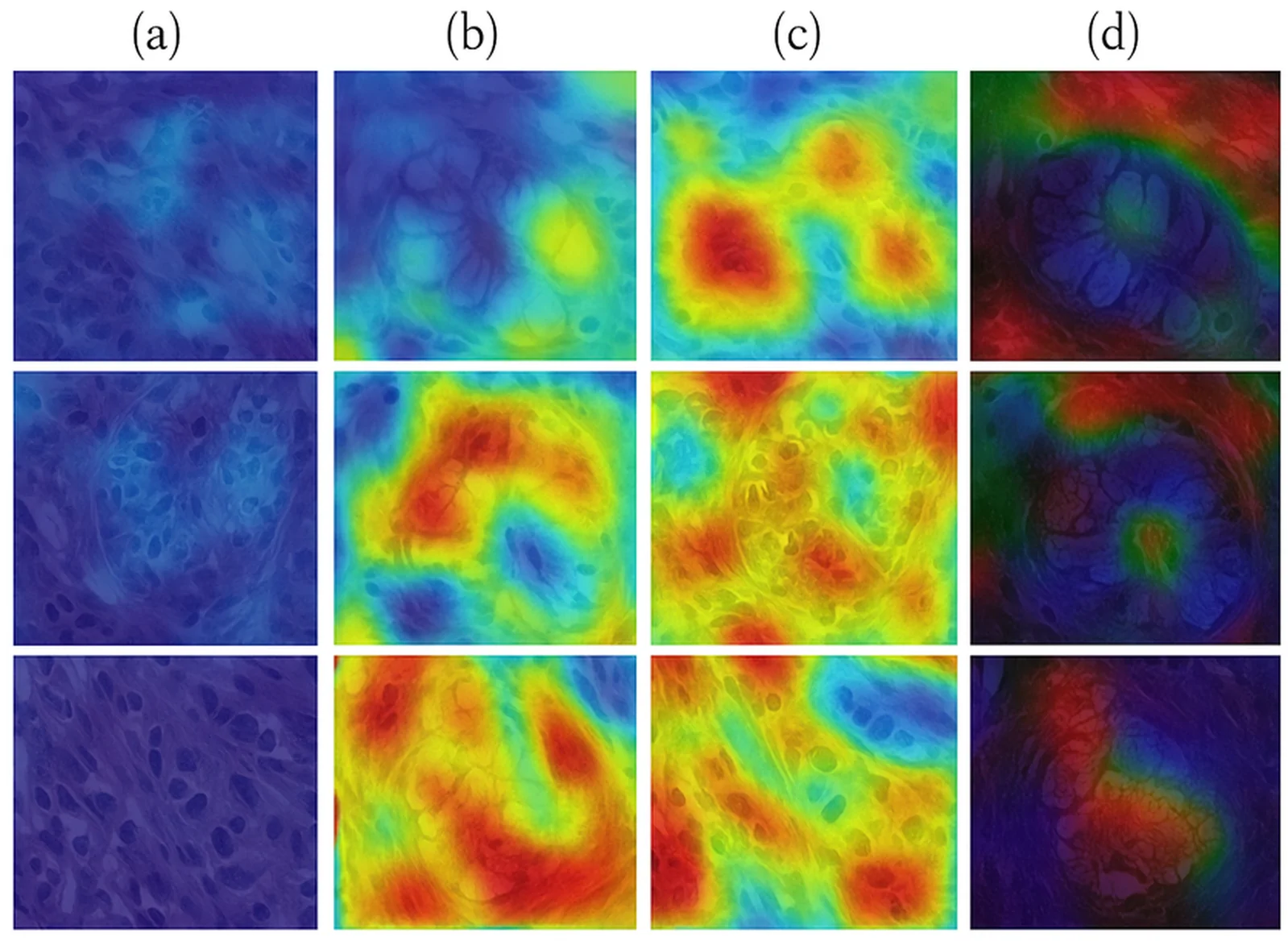
Representative Grad-CAM visualizations for EffAtt-LungNet. The heatmaps are illustrative qualitative examples of model responses and were not validated against pathologist annotations.

**Fig 6:**
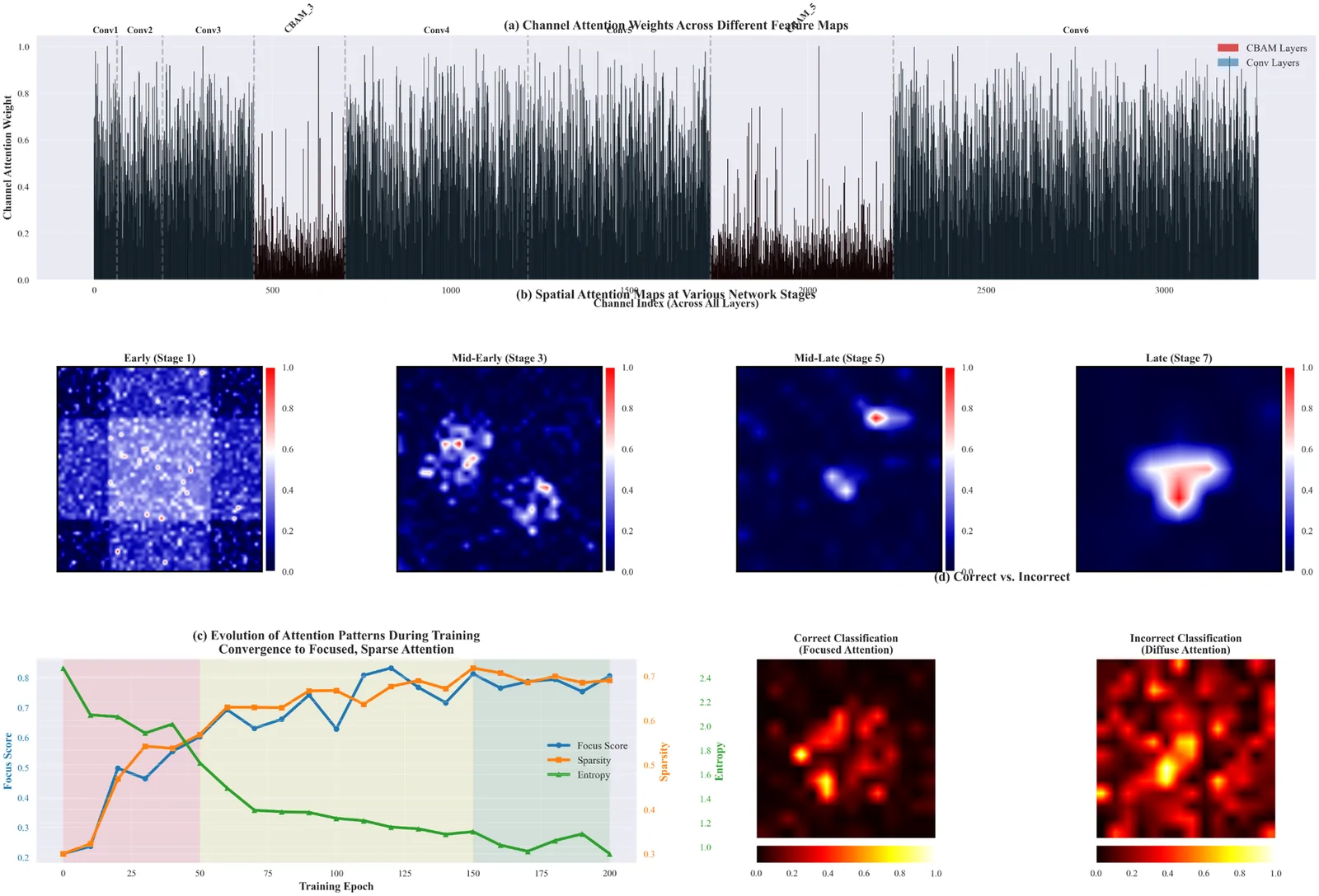
Representative CBAM response maps for EffAtt-LungNet. (a) input tile; (b) channel-refined response; (c) spatial attention map; and (d) overlay on the input tile. Warmer colours indicate higher model-derived attention weights and do not represent pathologist-validated regions of diagnostic importance.

Several residual errors occurred between LUAD and LUSC, and the corresponding maps highlighted visually plausible morphology-associated regions. In the absence of pathologist annotation, these responses cannot be assigned to specific diagnostic structures. The maps should therefore be interpreted only as qualitative representations of model behaviour rather than expert-validated localization of diagnostic features.

### Comparison with prior work

The proposed model is competitive with recent histopathology-focused methods and adds an internal ablation and a qualitative interpretability analysis. Direct rank ordering across studies should be avoided unless datasets, preprocessing, class definitions, and evaluation protocols are closely matched.

As summarised in Table 13, the proposed model produced results within the range reported by recent histopathology methods and includes an internal ablation and qualitative interpretation. Direct ranking is not appropriate because the studies differ in dataset provenance, preprocessing, augmentation, classes, partitioning, evaluation units, and treatment of related images. The comparison is therefore contextual.

**Table 13:** Contextual comparison with selected prior studies. Results are not directly rank-comparable because the studies differ in dataset provenance and version, preprocessing, augmentation, class definitions, partitioning strategy, evaluation unit, and possible source-image dependence.

Study	Method	Dataset	Classes	Accuracy
Poornima and Anand (2024)	CNN + augmentation	Private	–	97.74%
Illakiya et al. (2025)	DenseNet169 + CBAM	Histopathology	3	99.7%
Imran et al. (2024)	CNN + ViT	LC25000	3	98.8%
Kumar and Nelson (2025)	EfficientNetB3	Histopathology	3	99.93%
**EffAtt-LungNet (Ours)**	**EfficientNetB3 + CBAM**	**LC25000-derived augmented lung corpus**	**3**	**99.86%**

## Discussion

EffAtt-LungNet produced a modest improvement over the EfficientNetB3 baseline with a limited increase in computational cost. Residual errors occurred mainly between LUAD and LUSC. These findings apply to the image-level LC25000-derived benchmark and cannot be interpreted as evidence of performance on independent clinical material.

The architectural ablation showed increasing benchmark performance when CBAM was placed in the later EfficientNetB3 stages. This pattern is consistent with selective weighting of higher-level feature responses. The qualitative maps illustrate where the model assigned greater response weights but do not provide biological validation of those regions.

LC25000 is widely used for histopathology classification benchmarks, but its restricted acquisition setting and limited acquisition diversity do not represent the full variation encountered across routine pathology services. Differences in tissue processing, staining protocols, microscopes or scanners, image quality, institutional workflows, and patient populations can substantially alter image appearance and model performance. The present evaluation also used curated images rather than whole-slide images. It therefore does not address tissue and tumour heterogeneity across a slide, artefacts, variation in tumour content, selection of diagnostic regions, or aggregation of evidence across an entire specimen. The absence of patient- and slide-level identifiers prevents assessment at the patient or specimen level.

The released LC25000 images were derived from a smaller collection through augmentation. Although variants generated in the present study were grouped with their immediate parent images, independence among derivatives created during the original construction of LC25000 could not be verified. This unresolved dependence may inflate image-level test performance and is especially relevant in a near-ceiling setting. The reported gain over EfficientNetB3 should therefore be interpreted as a modest internal benchmark difference, not a demonstrated gain on independent clinical samples.

The Grad-CAM and CBAM maps provide only qualitative descriptions of model responses. Independent, multi-centre evaluation on non-augmented cohorts is needed to assess variation across tissue preparation, staining, imaging systems, and patient populations. Whole-slide and patient-level assessment and systematic comparison of attribution maps with pathologist annotations are also needed before clinical translation is considered. These requirements are consistent with broader developments in clinically oriented medical AI, where predictive performance is increasingly complemented by interpretability and evaluation across independent institutions. Recent studies have demonstrated robust and interpretable deep-learning frameworks using multi-centre imaging cohorts, including explainable prognostic models and multi-institutional digital-biopsy approaches that combine imaging predictions with methods such as Grad-CAM and SHAP [29,30]. Multi-centre radiomics-based virtual-biopsy studies have similarly illustrated the importance of evaluation across independent clinical institutions when developing non-invasive imaging biomarkers [31]. Although these studies address different diseases and imaging modalities from the present histopathology benchmark, they provide relevant methodological examples of how explainability and independent multi-institutional validation can be incorporated into clinically oriented AI frameworks. Future evaluation of EffAtt-LungNet should therefore extend beyond internal tile-level performance to independent multi-centre histopathology cohorts and systematic expert assessment of attribution maps

## Conclusion

This study introduced EffAtt-LungNet, an EfficientNetB3 model with CBAM-based feature refinement for three-class lung histopathology classification. On the LC25000-derived image-level test partition, the model reached 99.86% accuracy, precision, recall, and F1 score, with an AUC of 0.9997, while adding a limited computational cost relative to EfficientNetB3. The improvement over the baseline was modest and applies only to this internal benchmark.

These findings do not establish source-independent generalization or clinical validity because relationships among images created during the original LC25000 augmentation process could not be excluded. External evaluation on independent, non-augmented, multi-centre cohorts with documented variation in patient populations, staining protocols, and imaging systems is required before any translational or clinical claim can be made. Future work should also include whole-slide and patient-level evaluation, calibration analysis, and pathologist-based assessment of model attribution maps.

## References

[1] Patharia P, Sethy PK. LungCarcinoGrade-EffNetSVM: A novel approach to lung carcinoma grading using EfficientNetB0 and support vector machine. In: 2024 8th International Conference on I-SMAC (IoT in Social, Mobile, Analytics and Cloud). 2024. 1183–8.

[2] Imran M, Haq B, Elbasi E, Topcu AE, Shao W. Transformer-based hierarchical model for non-small cell lung cancer detection and classification. IEEE Access. 2024;12:145920–33. doi: 10.1109/access.2024.3449230

[3] Kanwal S, Ali S, Alahmadi TJ, Assam M, Ansari GJ, Bhatti UA. Pretrained model with CNN for lung cancer identification and classification. In: 2024 7th International Conference on Pattern Recognition and Artificial Intelligence (PRAI). 2024. 811–6. 10.1109/prai62207.2024.10826706

[4] Hasanuzzaman Md. Lung cancer classification optimization using CNN and edge detector from CT scan images. In: 2024 IEEE 15th Annual ubiquitous computing, electronics mobile communication conference (UEMCON), 2024. 1–6. 10.1109/uemcon62879.2024.10754686

[5] Wehbe A, Dellepiane S, Minetti I. Enhanced lung cancer detection and TNM staging using YOLOv8 and TNMClassifier: An integrated deep learning approach for CT imaging. IEEE Access. 2024;12:141414–24. doi: 10.1109/access.2024.3462629

[6] Rani R, Gupta S. Enhanced chest cancer classification using convolutional neural networks on CT scan images for improved diagnostic accuracy. In: 2024 5th International conference on smart electronics and communication (ICOSEC). 2024. 1237–41. 10.1109/icosec61587.2024.10722449

[7] Sumithra B, Vallathan G, Raman Kumar M, Govindharaju K. Deep learning for accurate chest disease classification: A CNN-based approach for lung cancer subtypes and normal cells. In: 2023 International conference on system, computation, automation and networking (ICSCAN). 2023. 1–7. 10.1109/icscan58655.2023.10394855

[8] Illakiya T, Akhilesh S, Karthik DS. A hybrid deep learning model for lung cancer classification using DenseNet169 and convolutional block attention module. In: 2025 International conference on multi-agent systems for collaborative intelligence (ICMSCI), 2025. 983–8.

[9] Divya N, Dhilip P, Manish S, Abilash I. Deep learning based lung cancer prediction using CNN. In: 2024 International Conference on Signal Processing, Computation, Electronics, Power and Telecommunication (IConSCEPT). 2024. 1–4.

[10] Kumar A, Nelson L. Early detection and classification of lung cancer using an EfficientNetB3 deep learning model on histopathological images. In: 2025 International Conference on Multi-Agent Systems for Collaborative Intelligence (ICMSCI). 2025. 932–7. 10.1109/icmsci62561.2025.10894566

[11] Shimna PK, Shirly Edward A, Roshini TV. A review on diagnosis of lung cancer and lung nodules in histopathological images using deep convolutional neural network. In: 2023 International Conference on Artificial Intelligence and Applications (ICAIA) Alliance Technology Conference (ATCON-1), 2023. 1–4. 10.1109/icaia57370.2023.10169738

[12] Rehman A, Kashif M, Abunadi I, Ayesha N. Lung cancer detection and classification from chest CT scans using machine learning techniques. In: 2021 1st International Conference on Artificial Intelligence and Data Analytics (CAIDA), 2021. 101–4. 10.1109/caida51941.2021.9425269

[13] Eshan SH, Hasan RR, Howlader S, Iqbal A, Forhad S, Aliuzzaman S, et al. X band on-body antenna design for lung cancer detection using single-walled carbon nanotubes. In: 2023 8th International Conference on Robotics and Automation Engineering (ICRAE), 2023. 182–6. 10.1109/icrae59816.2023.10458599

[14] Vij A, Kaswan KS. Prediction of lung cancer using convolution neural networks. In: 2023 International Conference on Artificial Intelligence and Smart Communication (AISC). 2023. 737–41. 10.1109/aisc56616.2023.10085058

[15] Madhuvappam CA, Kumar DV, Kanna S, Vaishnodevi S, Murali G, Karthick M. Enhanced lung cancer detection using ensemble learning algorithms: a comparative study of LightGBM & CatBoost. In: 2024 5th International conference on image processing and capsule networks (ICIPCN). 2024. 324–8.

[16] V SK, Sonali CS, S CB, B JK, Easa M. CNN models comparison for lung cancer classification using CT and PET scans. In: 2022 IEEE 2nd Mysore Sub Section International Conference (MysuruCon). 2022. 1–5. 10.1109/mysurucon55714.2022.9972704

[17] G P, L A. Novel AI multimodal approach for combating against pulmonary carcinoma. In: 2024 5th International Conference for Emerging Technology (INCET). 2024. 1–6. 10.1109/incet61516.2024.10593234

[18] Sherin K, Jaspin K, S H, Sivanantham S, Senthilvelan S, Theerthagiri S. Optimising lung cancer screening through advanced techniques for image analysis: an efficient deep learning approach. In: 2023 4th International Conference on Smart Electronics and Communication (ICOSEC). 2023. 1528–35. 10.1109/icosec58147.2023.10275884

[19] A HA, M T, Rajamohanan R, M A. Deep residual network for effective lung cancer detection on computed tomography images. In: 2024 IEEE International Conference on Contemporary Computing and Communications (InC4). 2024. 1–6. 10.1109/inc460750.2024.10649053

[20] Rajaram R, Midhunchakkaravarthy, Selvam J, Vajravelu A. Enhancing lung cancer detection using VGG-16: Leveraging deep convolutional neural networks for accurate medical image analysis. In: 2024 2nd International Conference on Recent Trends in Microelectronics, Automation, Computing and Communications Systems (ICMACC). 2024. 190–4. 10.1109/icmacc62921.2024.10894308

[21] Ramya S, Minu RI, Magesh KT. An automated approach for identification of oral squamous cell carcinoma based on 2D-ICNN. In: 2023 Second International Conference on Augmented Intelligence and Sustainable Systems (ICAISS). 2023. 155–61. 10.1109/icaiss58487.2023.10250540

[22] K SK, V K, S P, V V. Lung – pleura carcinoma detection using machine learning. In: 2021 3rd International Conference on Signal Processing and Communication (ICPSC). 2021. 294–8. 10.1109/icspc51351.2021.9451769

[23] Kumar A, Nelson L, Venu VS. Detecting colon and lung cancer through deep learning CNN model. In: 2024 2nd International conference on computer, communication and control (IC4). 2024. 1–6. 10.1109/ic457434.2024.10486421

[24] Keerthi P, Iniyan S. Lung cancer classification using deep neural networks. In: 2025 AI-Driven Smart Healthcare for Society 5.0. 2025. 1–6. 10.1109/ieeeconf64992.2025.10963162

[25] Sallagundla B, Srungarapati RK, Dammu JBN. Vision transformer-based computer-aided diagnosis system for histopathological image classification in lung and colon cancer. In: 2024 International Conference on Computing and Intelligent Reality Technologies (ICCIRT). 2024. 43–8.

[26] Murthy Nimmagadda S, Likhitha K, Srilatha G, Sree SM. Lung cancer prediction and classification using machine learning algorithms. In: 2024 International Conference on Expert Clouds and Applications (ICOECA). 2024. 1012–5. 10.1109/icoeca62351.2024.00176

[27] Lathakumari KR, Ramachandra AC, Avanthi UC, Basil Ronald C, Bhavatharani T. Classification of non-small cell lung cancer using deep learning. In: 2023 International Conference on Applied Intelligence and Sustainable Computing (ICAISC), 2023. 1–5. 10.1109/icaisc58445.2023.10200012

[28] Borkowski AA, Bui MM, Thomas LB, Wilson CP, DeLand LA, Mastorides SM. Lung and colon cancer histopathological image dataset (LC25000). 2019. doi: 10.48550/arXiv.1912.12142

[29] Jiang C, Jiang Z, Zhang X, Qu L, Fu K, Teng Y, et al. Robust and interpretable deep learning system for prognostic stratification of extranodal natural killer/T-cell lymphoma. Eur J Nucl Med Mol Imaging. 2025;52(5):1739–50. doi: 10.1007/s00259-024-07024-x 39714634

[30] Jiang C, Jiang Z, Zhang Z, Huang H, Zhou H, Jiang Q, et al. An explainable transformer model integrating PET and tabular data for histologic grading and prognosis of follicular lymphoma: a multi-institutional digital biopsy study. Eur J Nucl Med Mol Imaging. 2025;52(7):2384–96. doi: 10.1007/s00259-025-07090-9 39883138

[31] Jiang C, Qian C, Jiang Q, Zhou H, Jiang Z, Teng Y, et al. Virtual biopsy for non-invasive identification of follicular lymphoma histologic transformation using radiomics-based imaging biomarker from PET/CT. BMC Med. 2025;23(1):49. doi: 10.1186/s12916-025-03893-7 39875864 PMC11776338

