# Supplementary material for: EffAtt-LungNet: An attention-guided EfficientNet framework for three-class lung histopathology classification
Source: PLoS One. 2026 Oct 5;21(10):e0359302. doi: 10.1371/journal.pone.0359302 (PMC13638055; doi:10.1371/journal.pone.0359302)
Supplement: Supplementary file 1 [file pone.0359302.s001.docx]

**Reviewer Comments for Manuscript PONE-D-26-38240**

**Title:**EffAtt-LungNet: an attention-guided EfficientNet framework for three-class lung histopathology classification

**General Assessment:**

This manuscript presents a well-structured study on the application of an attention-guided deep learning framework for three-class lung histopathology classification. The authors combine EfficientNetB3 with the Convolutional Block Attention Module and demonstrate strong performance on an augmented LC25000-derived dataset. The topic is timely and relevant to the computational pathology community. The experimental design is generally sound, and the authors are appropriately cautious in framing their results as benchmark-level findings rather than clinically ready tools.

I recommend **minor revisions**.

**Major Comments:**

**1. Discussion of dataset representativeness and generalisability**

The authors rightly emphasise that their results are not clinically deployable without external validation. I would encourage them to expand slightly on why the LC25000 dataset, despite its widespread use, may not fully reflect real-world histopathology practice. Aspects such as single-centre origin, limited stain and scanner variability, and the distinction between tile-level and slide-level diagnosis could be briefly addressed. A short paragraph in the Discussion would help readers interpret the reported performance with appropriate caution.

**2. Potential data leakage from augmentation**

The authors state that augmented variants derived from the same source image were assigned to the same partition, which is good practice. However, given that the original dataset contained only 5,000 images per class and was expanded to 8,500 through augmentation, I would suggest adding a brief note on whether any additional overlap check was performed between training and test sets, for example using image hashing or similarity metrics, to further rule out unintended information leakage.

**3. Positioning of interpretability analysis**

The Grad-CAM and CBAM visualisations are informative, and the authors acknowledge that they were not validated by a pathologist. I would suggest stating more explicitly in the main text that these visualisations are intended as illustrative examples of model behaviour rather than expert-validated localisation of diagnostic features. A brief mention in the Future Work section about involving clinical collaborators for systematic evaluation would also be welcome.

**4. Contextualisation of comparison with prior work**

Table 13 compares the proposed method with several previous studies, but these studies differ in dataset, preprocessing, class definitions, and evaluation protocols. The authors already caution against direct ranking, which is appropriate. I would suggest adding a short note in the table caption or accompanying text to highlight the main differences that affect comparability, so that readers can better appreciate the contextual nature of the comparison.

**Minor Comments:**

**Abstract and Conclusion:**The statement that external validation is required before any translational claim can be made is excellent and should be reiterated in the Conclusion section for emphasis.

**Equation presentation:**Equations (7)–(10) are somewhat dense. Consider adding a brief intuitive explanation after each to improve readability for readers less familiar with attention mechanisms.
