# Supplementary material for: EffAtt-LungNet: An attention-guided EfficientNet framework for three-class lung histopathology classification
Source: PLoS One. 2026 Oct 5;21(10):e0359302. doi: 10.1371/journal.pone.0359302 (PMC13638055; doi:10.1371/journal.pone.0359302)
Supplement: Peer review history

# Peer review: Decision Letter 0

**DOI:** 10.1371/journal.pone.0359302.r001

**Authors:** Chong Jiang

31 Aug 2026

Dear Dr. Kajla,

Thank you for submitting your manuscript to PLOS One. After careful consideration, we feel that it has merit but does not fully meet PLOS One’s publication criteria as it currently stands. Therefore, we invite you to submit a revised version of the manuscript that addresses the points raised during the review process.

Please submit your revised manuscript by Oct 30 2026 11:59PM. If you will need more time than this to complete your revisions, please reply to this message or contact the journal office at plosone@plos.org. When you're ready to submit your revision, log on to https://www.editorialmanager.com/pone/ and select the 'Submissions Needing Revision' folder to locate your manuscript file.

- A letter that responds to each point raised by the academic editor and reviewer(s). You should upload this letter as a separate file labeled 'Response to Reviewers'.
- A marked-up copy of your manuscript that highlights changes made to the original version. You should upload this as a separate file labeled 'Revised Manuscript with Track Changes'.
- An unmarked version of your revised paper without tracked changes. You should upload this as a separate file labeled 'Manuscript'.

If you would like to make changes to your financial disclosure, please include your updated statement in your cover letter. Guidelines for resubmitting your figure files are available below the reviewer comments at the end of this letter.

If applicable, we recommend that you deposit your laboratory protocols in protocols.io to enhance the reproducibility of your results. Protocols.io assigns your protocol its own identifier (DOI) so that it can be cited independently in the future. For instructions see: https://journals.plos.org/plosone/s/submission-guidelines#loc-laboratory-protocols. Additionally, PLOS One offers an option for publishing peer-reviewed Lab Protocol articles, which describe protocols hosted on protocols.io. Read more information on sharing protocols at https://plos.org/protocols?utm_medium=editorial-email&utm_source=authorletters&utm_campaign=protocols.

As the corresponding author, your ORCID iD is verified in the submission system and will appear in the published article. PLOS supports the use of ORCID, and we encourage all coauthors to register for an ORCID iD and use it as well. Please encourage your coauthors to verify their ORCID iD within the submission system before final acceptance, as unverified ORCID iDs will not appear in the published article. *Only*  the individual author can complete the verification step; PLOS staff *cannot*  verify ORCID iDs on behalf of authors.

We look forward to receiving your revised manuscript.

Kind regards,

Chong Jiang

Academic Editor

PLOS One

Journal Requirements:

When submitting your revision, we need you to address these additional requirements.

1. Please ensure that your manuscript meets PLOS ONE's style requirements, including those for file naming. The PLOS ONE style templates can be found at

https://journals.plos.org/plosone/s/file?id=wjVg/PLOSOne_formatting_sample_main_body.pdf and

https://journals.plos.org/plosone/s/file?id=ba62/PLOSOne_formatting_sample_title_authors_affiliations.pdf

2. Please note that PLOS One has specific guidelines on code sharing for submissions in which author-generated code underpins the findings in the manuscript. In these cases, we expect all author-generated code to be made available without restrictions upon publication of the work. Please review our guidelines at https://journals.plos.org/plosone/s/materials-and-software-sharing#loc-sharing-code and ensure that your code is shared in a way that follows best practice and facilitates reproducibility and reuse.

3. Please note that your Data Availability Statement is currently missing the repository name and/or the DOI/accession number of each dataset OR a direct link to access each database. If your manuscript is accepted for publication, you will be asked to provide these details on a very short timeline. We therefore suggest that you provide this information now, though we will not hold up the peer review process if you are unable.

4. If the reviewer comments include a recommendation to cite specific previously published works, please review and evaluate these publications to determine whether they are relevant and should be cited. There is no requirement to cite these works unless the editor has indicated otherwise.

Additional Editor Comments:

Dear Authors,

Thank you for submitting your manuscript.

After careful consideration of the manuscript and the reviewers’ assessments, I have decided that a major revision is required before the manuscript can be considered further.

The study is well structured and addresses a relevant methodological question in computational pathology. The source-aware splitting strategy, multi-seed analysis, ablation experiments, statistical comparison, and appropriately cautious positioning of the model as a tile-level benchmark are strengths.

However, an important issue concerning the provenance of the LC25000 images and the independence of the training and test sets must be resolved. Because the publicly released LC25000 dataset itself contains augmented derivatives of a smaller set of original histopathology images, it is essential to demonstrate that related derivatives of the same underlying source tissue image did not occur across different data partitions. Please address this issue carefully and, if necessary, repeat the analysis using a leakage-free split defined at the earliest identifiable source-image level.

The remaining reviewer comments regarding methodological reproducibility, interpretation of the modest improvement over the strong baseline, qualitative explainability, dataset generalizability, and clinical framing should also be addressed carefully.

Please provide a detailed point-by-point response and clearly identify any additional analyses and corresponding revisions.

Sincerely,

Academic Editor

PLOS ONE

Reviewer 1

General Assessment:

This manuscript presents a well-structured study on the application of an attention-guided deep learning framework for three-class lung histopathology classification. The authors combine EfficientNetB3 with the Convolutional Block Attention Module and demonstrate strong performance on an augmented LC25000-derived dataset. The topic is timely and relevant to the computational pathology community. The experimental design is generally sound, and the authors are appropriately cautious in framing their results as benchmark-level findings rather than clinically ready tools.

Major Comments:

1. Discussion of dataset representativeness and generalisability

The authors rightly emphasise that their results are not clinically deployable without external validation. I would encourage them to expand slightly on why the LC25000 dataset, despite its widespread use, may not fully reflect real-world histopathology practice. Aspects such as single-centre origin, limited stain and scanner variability, and the distinction between tile-level and slide-level diagnosis could be briefly addressed. A short paragraph in the Discussion would help readers interpret the reported performance with appropriate caution.

2. Potential data leakage from augmentation

The authors state that augmented variants derived from the same source image were assigned to the same partition, which is good practice. However, given that the original dataset contained only 5,000 images per class and was expanded to 8,500 through augmentation, I would suggest adding a brief note on whether any additional overlap check was performed between training and test sets, for example using image hashing or similarity metrics, to further rule out unintended information leakage.

3. Positioning of interpretability analysis

The Grad-CAM and CBAM visualisations are informative, and the authors acknowledge that they were not validated by a pathologist. I would suggest stating more explicitly in the main text that these visualisations are intended as illustrative examples of model behaviour rather than expert-validated localisation of diagnostic features. A brief mention in the Future Work section about involving clinical collaborators for systematic evaluation would also be welcome.

4. Contextualisation of comparison with prior work

Table 13 compares the proposed method with several previous studies, but these studies differ in dataset, preprocessing, class definitions, and evaluation protocols. The authors already caution against direct ranking, which is appropriate. I would suggest adding a short note in the table caption or accompanying text to highlight the main differences that affect comparability, so that readers can better appreciate the contextual nature of the comparison.

Minor Comments:

Abstract and Conclusion: The statement that external validation is required before any translational claim can be made is excellent and should be reiterated in the Conclusion section for emphasis.

Equation presentation: Equations (7)–(10) are somewhat dense. Consider adding a brief intuitive explanation after each to improve readability for readers less familiar with attention mechanisms.

Reviewer 2

This is a well-structured benchmark study evaluating an attention-guided EfficientNet framework for three-class lung histopathology classification. The source-aware splitting strategy, multi-seed analysis, architectural ablation, computational-efficiency evaluation, and cautious positioning of the model as a tile-level benchmark are strengths. I have several comments that should be addressed to further improve methodological transparency and interpretation.

1. The provenance of the LC25000 images and the definition of the “source image” require important clarification. The manuscript treats the 5,000 images per LC25000 class as the original images and subsequently generates additional augmented variants. However, the publicly released LC25000 dataset itself was created by augmenting a much smaller set of original histopathology images. Please clarify whether the source-aware split used here traces each released LC25000 image back to its pre-augmentation original image, rather than merely grouping the additional augmentations generated in the present study. If this deeper provenance was available, please describe the mapping explicitly and confirm that all derivatives of the same original tissue image were confined to one partition. The phrase “original, non-augmented LC25000 lung tiles” should also be corrected accordingly. This clarification is particularly important given the near-ceiling test performance.

2. Please provide the complete hyperparameter settings for the composite loss. The Methods specify cross-entropy plus focal loss and L2 regularization, but the final values of λf, λr, and αj, as well as the ranges considered during validation-based tuning, are not clearly reported. Since the classes are already balanced, it would also be useful to briefly explain why adding focal loss materially improves optimization and whether the same settings were used across all five random-seed experiments.

3. The statistical comparison with EfficientNetB3 could be presented a little more completely. McNemar’s test supports a difference in paired classification errors, but reporting a confidence interval for the absolute improvement in accuracy or macro-F1 would make the magnitude of the gain easier to interpret. Given the already very high EfficientNetB3 performance, the current wording should continue to emphasize a modest benchmark improvement rather than a large practical gain.

4. The Grad-CAM and CBAM visualizations are useful, but the interpretation should remain strictly qualitative. The manuscript appropriately acknowledges that the maps were neither pathologist-annotated nor quantitatively evaluated; however, statements that attention focused specifically on “glandular lumina,” “keratin pearls,” or other diagnostically relevant structures may still imply stronger biological validation than was actually performed. I suggest using wording such as “visually plausible morphology-associated regions” unless expert pathological assessment is provided.

5. The Discussion could further place the study within the broader methodological literature on robust and interpretable medical AI, digital biopsy, and independent multicenter validation. This is particularly relevant because the present study demonstrates excellent internal benchmark performance but appropriately recognizes that external validation is required before clinical translation. Related methodological studies may provide useful context, for example DOI: 10.1007/s00259-024-07024-x, DOI: 10.1007/s00259-025-07090-9, and DOI: 10.1186/s12916-025-03893-7. These studies could be discussed alongside other relevant literature to illustrate how explainability and independent multi-institutional validation are increasingly incorporated into clinically oriented AI frameworks.

Overall, the manuscript is technically interesting and potentially suitable for publication after minor revision, provided that the LC25000 source-image provenance and data-splitting procedure are clearly resolved.

[Note: HTML markup is below. Please do not edit.]

Reviewers' comments:

Reviewer's Responses to Questions

**Comments to the Author**

1. Is the manuscript technically sound, and do the data support the conclusions?

Reviewer #1: Yes

Reviewer #2: Yes

**********

2. Has the statistical analysis been performed appropriately and rigorously?

Reviewer #1: Yes

Reviewer #2: Yes

**********

3. Have the authors made all data underlying the findings in their manuscript fully available?

The PLOS Data policy

Reviewer #1: Yes

Reviewer #2: Yes

**********

4. Is the manuscript presented in an intelligible fashion and written in standard English?

Reviewer #1: Yes

Reviewer #2: Yes

**********

Reviewer #1: Reviewer Comments for Manuscript PONE-D-26-38240

Title:EffAtt-LungNet: an attention-guided EfficientNet framework for three-class lung histopathology classification

General Assessment:

This manuscript presents a well-structured study on the application of an attention-guided deep learning framework for three-class lung histopathology classification. The authors combine EfficientNetB3 with the Convolutional Block Attention Module and demonstrate strong performance on an augmented LC25000-derived dataset. The topic is timely and relevant to the computational pathology community. The experimental design is generally sound, and the authors are appropriately cautious in framing their results as benchmark-level findings rather than clinically ready tools.

I recommend minor revisions.

Major Comments:

1. Discussion of dataset representativeness and generalisability

The authors rightly emphasise that their results are not clinically deployable without external validation. I would encourage them to expand slightly on why the LC25000 dataset, despite its widespread use, may not fully reflect real-world histopathology practice. Aspects such as single-centre origin, limited stain and scanner variability, and the distinction between tile-level and slide-level diagnosis could be briefly addressed. A short paragraph in the Discussion would help readers interpret the reported performance with appropriate caution.

2. Potential data leakage from augmentation

The authors state that augmented variants derived from the same source image were assigned to the same partition, which is good practice. However, given that the original dataset contained only 5,000 images per class and was expanded to 8,500 through augmentation, I would suggest adding a brief note on whether any additional overlap check was performed between training and test sets, for example using image hashing or similarity metrics, to further rule out unintended information leakage.

3. Positioning of interpretability analysis

The Grad-CAM and CBAM visualisations are informative, and the authors acknowledge that they were not validated by a pathologist. I would suggest stating more explicitly in the main text that these visualisations are intended as illustrative examples of model behaviour rather than expert-validated localisation of diagnostic features. A brief mention in the Future Work section about involving clinical collaborators for systematic evaluation would also be welcome.

4. Contextualisation of comparison with prior work

Table 13 compares the proposed method with several previous studies, but these studies differ in dataset, preprocessing, class definitions, and evaluation protocols. The authors already caution against direct ranking, which is appropriate. I would suggest adding a short note in the table caption or accompanying text to highlight the main differences that affect comparability, so that readers can better appreciate the contextual nature of the comparison.

Minor Comments:

Abstract and Conclusion:The statement that external validation is required before any translational claim can be made is excellent and should be reiterated in the Conclusion section for emphasis.

Equation presentation:Equations (7)–(10) are somewhat dense. Consider adding a brief intuitive explanation after each to improve readability for readers less familiar with attention mechanisms.

Reviewer #2: This is a well-structured benchmark study evaluating an attention-guided EfficientNet framework for three-class lung histopathology classification. The source-aware splitting strategy, multi-seed analysis, architectural ablation, computational-efficiency evaluation, and cautious positioning of the model as a tile-level benchmark are strengths. I have several comments that should be addressed to further improve methodological transparency and interpretation.

1. The provenance of the LC25000 images and the definition of the “source image” require important clarification. The manuscript treats the 5,000 images per LC25000 class as the original images and subsequently generates additional augmented variants. However, the publicly released LC25000 dataset itself was created by augmenting a much smaller set of original histopathology images. Please clarify whether the source-aware split used here traces each released LC25000 image back to its pre-augmentation original image, rather than merely grouping the additional augmentations generated in the present study. If this deeper provenance was available, please describe the mapping explicitly and confirm that all derivatives of the same original tissue image were confined to one partition. The phrase “original, non-augmented LC25000 lung tiles” should also be corrected accordingly. This clarification is particularly important given the near-ceiling test performance.

2. Please provide the complete hyperparameter settings for the composite loss. The Methods specify cross-entropy plus focal loss and L2 regularization, but the final values of λf, λr, and αj, as well as the ranges considered during validation-based tuning, are not clearly reported. Since the classes are already balanced, it would also be useful to briefly explain why adding focal loss materially improves optimization and whether the same settings were used across all five random-seed experiments.

3. The statistical comparison with EfficientNetB3 could be presented a little more completely. McNemar’s test supports a difference in paired classification errors, but reporting a confidence interval for the absolute improvement in accuracy or macro-F1 would make the magnitude of the gain easier to interpret. Given the already very high EfficientNetB3 performance, the current wording should continue to emphasize a modest benchmark improvement rather than a large practical gain.

4. The Grad-CAM and CBAM visualizations are useful, but the interpretation should remain strictly qualitative. The manuscript appropriately acknowledges that the maps were neither pathologist-annotated nor quantitatively evaluated; however, statements that attention focused specifically on “glandular lumina,” “keratin pearls,” or other diagnostically relevant structures may still imply stronger biological validation than was actually performed. I suggest using wording such as “visually plausible morphology-associated regions” unless expert pathological assessment is provided.

5. The Discussion could further place the study within the broader methodological literature on robust and interpretable medical AI, digital biopsy, and independent multicenter validation. This is particularly relevant because the present study demonstrates excellent internal benchmark performance but appropriately recognizes that external validation is required before clinical translation. Related methodological studies may provide useful context, for example DOI: 10.1007/s00259-024-07024-x, DOI: 10.1007/s00259-025-07090-9, and DOI: 10.1186/s12916-025-03893-7. These studies could be discussed alongside other relevant literature to illustrate how explainability and independent multi-institutional validation are increasingly incorporated into clinically oriented AI frameworks. ([PubMed][2])

Overall, the manuscript is technically interesting and potentially suitable for publication after minor revision, provided that the LC25000 source-image provenance and data-splitting procedure are clearly resolved.

**********

what does this mean?). If published, this will include your full peer review and any attached files.

If you choose “no”, your identity will remain anonymous but your review may still be made public.

**Do you want your identity to be public for this peer review?**  For information about this choice, including consent withdrawal, please see our Privacy Policy

Reviewer #1: No

Reviewer #2: No

**********

[NOTE: If reviewer comments were submitted as an attachment file, they will be attached to this email and accessible via the submission site. Please log into your account, locate the manuscript record, and check for the action link "View Attachments". If this link does not appear, there are no attachment files.]

To ensure your figures meet our technical requirements, please review our figure guidelines: https://journals.plos.org/plosone/s/figures

You may also use PLOS’s free figure tool, NAAS, to help you prepare publication quality figures: https://journals.plos.org/plosone/s/figures#loc-tools-for-figure-preparation.

NAAS will assess whether your figures meet our technical requirements by comparing each figure against our figure specifications.

Attachment Submitted filename: Minor revise.docx

# Author comment: Author response to Decision Letter 1

**DOI:** 10.1371/journal.pone.0359302.r002

10 Sep 2026

Response to the Reviewers comments:

Title: EffAtt-LungNet: an attention-guided EfficientNet framework for three-class lung histopathology classification

Reviewer 1

General Assessment:

This manuscript presents a well-structured study on the application of an attention-guided deep learning framework for three-class lung histopathology classification. The authors combine EfficientNetB3 with the Convolutional Block Attention Module and demonstrate strong performance on an augmented LC25000-derived dataset. The topic is timely and relevant to the computational pathology community. The experimental design is generally sound, and the authors are appropriately cautious in framing their results as benchmark-level findings rather than clinically ready tools.

Response: We thank the reviewer for the positive and constructive assessment of our manuscript. We appreciate the reviewer’s recognition of the relevance of the study, the overall experimental design, and the cautious interpretation of the findings. We have intentionally positioned EffAtt-LungNet as a controlled benchmark-level evaluation of attention-guided lung histopathology classification rather than as a clinically deployable diagnostic system. This scope is stated explicitly in the Introduction (page 2, lines 23-33), where the study is described as a controlled evaluation of tile-level classification under a defined experimental protocol and not as a claim of clinical readiness. We appreciate the reviewer’s acknowledgement of this framing.

Major Comments:

1. Discussion of dataset representativeness and generalisability

The authors rightly emphasise that their results are not clinically deployable without external validation. I would encourage them to expand slightly on why the LC25000 dataset, despite its widespread use, may not fully reflect real-world histopathology practice. Aspects such as single-centre origin, limited stain and scanner variability, and the distinction between tile-level and slide-level diagnosis could be briefly addressed. A short paragraph in the Discussion would help readers interpret the reported performance with appropriate caution.

Response: We thank the reviewer for this important suggestion. We have expanded the Discussion to clarify why the LC25000 dataset, although widely used as a histopathology benchmark, does not fully capture the variability encountered in routine pathology practice. Specifically, we now discuss potential variation in tissue processing, staining protocols, microscopes and scanners, image quality, institutions, and patient populations. We also clarify the distinction between classification of curated image tiles and whole-slide or patient-level diagnosis, including the limitations arising from the absence of slide- and patient-level identifiers. These additions are provided in the Discussion on page 14, lines 369-390. We also explicitly state in the Methods that the present task is a tile-level three-class classification problem rather than a slide-level or patient-level diagnostic task (page 4, lines 125–127). These revisions are intended to ensure that the reported near-ceiling benchmark performance is interpreted with appropriate caution and is not taken as evidence of real-world clinical generalisability.

2. Potential data leakage from augmentation

The authors state that augmented variants derived from the same source image were assigned to the same partition, which is good practice. However, given that the original dataset contained only 5,000 images per class and was expanded to 8,500 through augmentation, I would suggest adding a brief note on whether any additional overlap check was performed between training and test sets, for example using image hashing or similarity metrics, to further rule out unintended information leakage.

Response: We thank the reviewer for highlighting this important aspect of the experimental design. To prevent information leakage arising from the augmentation performed in the present study, partitioning was controlled at the parent-image level. Each publicly released LC25000 image and all additional fixed variants generated from that image were retained within the same train, validation, or test partition. Therefore, no parent image and its corresponding augmented variants generated in this study were distributed across different partitions.

In addition, stochastic training-time augmentation was applied exclusively to the training partition, whereas validation and test images were processed deterministically without random augmentation. This ensured that the validation and test sets were not exposed to augmentation generated during model optimisation.

We have clarified this leakage-control procedure in the revised manuscript in the Dataset description and splitting protocol (page 4, lines 117-123) and Data preprocessing section (page 4-5, lines 130-158). These measures ensure that the additional augmentation introduced in the present study does not create train-test overlap.

3. Positioning of interpretability analysis

The Grad-CAM and CBAM visualisations are informative, and the authors acknowledge that they were not validated by a pathologist. I would suggest stating more explicitly in the main text that these visualisations are intended as illustrative examples of model behaviour rather than expert-validated localisation of diagnostic features. A brief mention in the Future Work section about involving clinical collaborators for systematic evaluation would also be welcome.

Response: We thank the reviewer for this helpful suggestion. We have revised the interpretability analysis to make the intended role of the Grad-CAM and CBAM visualisations explicit. In the revised manuscript, these maps are described only as illustrative qualitative representations of model behaviour and not as expert-validated localisation of diagnostic features. We further clarify that the visualisations were not compared with pathologist annotations and that no localisation or attribution metric was calculated. These clarifications are provided in the Qualitative interpretation section on page 13, lines 342-346.

We have also strengthened the discussion of future validation. The revised Discussion states that systematic comparison of attribution maps with pathologist annotations is required before clinical translation (page 14, lines 390-394). In addition, the Conclusion identifies pathologist-based assessment of model attribution maps as an important direction for future work (page 15, lines 422-423).

4. Contextualisation of comparison with prior work

Table 13 compares the proposed method with several previous studies, but these studies differ in dataset, preprocessing, class definitions, and evaluation protocols. The authors already caution against direct ranking, which is appropriate. I would suggest adding a short note in the table caption or accompanying text to highlight the main differences that affect comparability, so that readers can better appreciate the contextual nature of the comparison.

Response: We thank the reviewer for this helpful suggestion. We have revised the caption and accompanying text of Table 13 to emphasise that the comparison with prior studies is contextual rather than a direct ranking. The revised table caption now explicitly notes that the studies differ in dataset provenance and version, preprocessing, augmentation, class definitions, partitioning strategy, evaluation unit, and possible source-image dependence (page 13, lines 348-351). We further reinforce this point in the accompanying text, where we state that direct ranking is not appropriate because of these methodological differences (page 13, lines 354-362). These additions are intended to help readers interpret the reported results within the appropriate experimental context.

Minor Comments:

Abstract and Conclusion: The statement that external validation is required before any translational claim can be made is excellent and should be reiterated in the Conclusion section for emphasis.

Response: We thank the reviewer for this important suggestion. We have reiterated the external-validation requirement in the Conclusion to ensure that the scope and limitations of the present study remain clear. The revised Conclusion now states that the findings do not establish source-independent generalisation or clinical validity and that external evaluation on independent, non-augmented, multi-centre cohorts is required before any translational or clinical claim can be made (page 14, lines 417-423). This is consistent with the corresponding statement already included in the Abstract.

Equation presentation: Equations (7)–(10) are somewhat dense. Consider adding a brief intuitive explanation after each to improve readability for readers less familiar with attention mechanisms.

Response: We thank the reviewer for this helpful suggestion. To improve the readability of the CBAM formulation for readers less familiar with attention mechanisms, we have added brief intuitive explanations after the channel- and spatial-attention equations. Specifically, after Equations (7)-(8), we explain that channel attention assigns greater weight to feature channels that contribute more strongly to the current representation, while reducing the influence of less responsive channels. After Equations (9)-(10), we explain that spatial attention determines which locations within the channel-refined feature map receive greater emphasis. We also clarify that channel attention refines which feature responses are retained, whereas spatial attention refines where those responses are weighted most strongly. These additions appear on page 6, lines 175-189.

Reviewer 2

This is a well-structured benchmark study evaluating an attention-guided EfficientNet framework for three-class lung histopathology classification. The source-aware splitting strategy, multi-seed analysis, architectural ablation, computational-efficiency evaluation, and cautious positioning of the model as a tile-level benchmark are strengths. I have several comments that should be addressed to further improve methodological transparency and interpretation.

Response: We thank the reviewer for the positive and constructive assessment of our manuscript. We appreciate the reviewer’s recognition of the methodological strengths of the study, including the controlled partitioning of additional augmented variants at the immediate-parent image level, the multi-seed stability analysis, architectural ablation experiments, computational-efficiency evaluation, and the cautious positioning of EffAtt-LungNet as an internal tile-level benchmark rather than a clinically deployable diagnostic system.

1. The provenance of the LC25000 images and the definition of the “source image” require important clarification. The manuscript treats the 5,000 images per LC25000 class as the original images and subsequently generates additional augmented variants. However, the publicly released LC25000 dataset itself was created by augmenting a much smaller set of original histopathology images. Please clarify whether the source-aware split used here traces each released LC25000 image back to its pre-augmentation original image, rather than merely grouping the additional augmentations generated in the present study. If this deeper provenance was available, please describe the mapping explicitly and confirm that all derivatives of the same original tissue image were confined to one partition. The phrase “original, non-augmented LC25000 lung tiles” should also be corrected accordingly. This clarification is particularly important given the near-ceiling test performance.

Response: We thank the reviewer for this important clarification. We agree that referring to the 5,000 images per class in the publicly distributed LC25000 lung subset as “original images” could incorrectly imply that these images represent independently acquired, pre-augmentation histopathology images.

We have therefore revised the terminology throughout the manuscript and now refer to these samples as “publicly released LC25000 images” or “released LC25000 images.” We also clarify that the released LC25000 dataset was itself constructed from a smaller underlying image collection through cropping and augmentation (page 4, lines 107–110).

For the augmentation performed specifically in the present study, partitioning was controlled at the immediate-parent image level. Each additional fixed variant generated from a released LC25000 image was retained in the same training, validation, or test partition as its immediate parent image (page 4, lines 111-117). Thus, the additional augmentation introduced in this study did not distribute a released parent image and its newly generated derivatives across different partitions.

To avoid overstating the level of provenance represented by this grouping procedure, we have also revised the manuscript so that the experimental setting is consistently described as an LC25000-derived image/tile-level benchmark, and the reported near-ceiling performance is interpreted within that controlled benchmark context rather than as evidence of source-independent or clinical generalisation. This limitation is reiterated in the Discussion (page 14, lines 383-389). The phrase “original, non-augmented LC25000 lung tiles” has also been removed and replaced with terminology that accurately refers to the publicly released LC25000 images.

2. Please provide the complete hyperparameter settings for the composite loss. The Methods specify cross-entropy plus focal loss and L2 regularization, but the final values of λf, λr, and αj, as well as the ranges considered during validation-based tuning, are not clearly reported. Since the classes are already balanced, it would also be useful to briefly explain why adding focal loss materially improves optimization and whether the same settings were used across all five random-seed experiments.

Response: We thank the reviewer for this important comment regarding the composite training objective. We have revised the Training Objective subsection to clarify the role and use of each component of the loss. The focal-loss focusing parameter was fixed at γ =2, and the remaining loss weights were selected using validation macro-F1 as the model-selection criterion. Importantly, because the three classes are numerically balanced, focal loss was not introduced to compensate for class-frequency imbalance. Instead, it was used to reduce the relative contribution of already well-classified examples and to place greater emphasis on difficult or ambiguous samples during optimization. This clarification is provided on page 7, lines 203-207.

We have also clarified that the selected composite-loss configuration was kept fixed across all five random-seed experiments and was not re-tuned separately for individual seeds (page 7, line 207). In addition, the loss-function ablation provides empirical support for including focal loss: relative to cross-entropy alone, the cross-entropy-plus-focal-loss configuration improved accuracy from 99.34% to 99.67%, reduced convergence from 147 to 126 epochs, and lowered validation loss from 0.0267 to 0.0203 (page 12, lines 333-335; page 13, Table 12, lines 336-340). These results indicate that the contribution of focal loss was primarily related to optimization of difficult examples rather than correction of class imbalance. To avoid introducing post-hoc numerical settings that cannot be independently verified from the retained experimental record, we have limited the revision to parameters and procedures that can be reported with confidence and have made this limitation explicit in the Methods.

3. The statistical comparison with EfficientNetB3 could be presented a little more completely. McNemar’s test supports a difference in paired classification errors, but reporting a confidence interval for the absolute improvement in accuracy or macro-F1 would make the magnitude of the gain easier to interpret. Given the already very high EfficientNetB3 performance, the current wording should continue to emphasize a modest benchmark improvement rather than a large practical gain.

Response: We thank the reviewer for this useful suggestion. We agree that statistical significance and effect magnitude should be distinguished clearly, particularly in a near-ceiling benchmark setting. We have therefore revised the Results to emphasize that the improvement over EfficientNetB3 is modest in absolute terms. EffAtt-LungNet improved accuracy from 98.97% to 99.86%, corresponding to an absolute gain of 0.89 percentage points, while McNemar’s test indicates a statistically significant difference in paired classification outcomes (page 10, lines 271–277).

We have also clarified that this statistical significance should not be interpreted as evidence of a large practical or clinical effect, because the EfficientNetB3 baseline already performs near the ceiling of this image-level benchmark. The manuscript therefore consistently characterizes the result as a modest internal benchmark improvement rather than a large practical gain.

The revised manuscript additionally reports bootstrap 95% confidence intervals for the principal EffAtt-LungNet performance measures in Table 7 (page 11, lines 292-297). We note, however, that these intervals characterize uncertainty in the model performance estimates themselves and are distinct from a confidence interval for the paired absolute difference between EffAtt-LungNet and EfficientNetB3. We have therefore avoided presenting a retrospectively reconstructed paired-difference interval.

4. The Grad-CAM and CBAM visualizations are useful, but the interpretation should remain strictly qualitative. The manuscript appropriately acknowledges that the maps were neither pathologist-annotated nor quantitatively evaluated; however, statements that attention focused specifically on “glandular lumina,” “keratin pearls,” or other diagnostically relevant structures may still imply stronger biological validation than was actually performed. I suggest using wording such as “visually plausible morphology-associated regions” unless expert pathological assessment is provided.

Response: We thank the reviewer for this important clarification. We agree that, in the absence of pathologist annotation or quantitative localisation assessment, the Grad-CAM and CBAM visualisations should remain strictly qualitative and should not be interpreted as biological or pathological validation of specific diagnostic structures.

Accordingly, we have revised the qualitative interpretation throughout the manuscript and removed statements assigning highlighted regions to specific structures such as glandular lumina, keratin pearls, or other named histopathological features. The revised text now describes the observed responses more conservatively as “visually plausible morphology-associated regions” (page 13, lines 342-344). We further state explicitly that the maps were not compared with pathologist annotations, that no localisation or attribution metric was calculated, and that the responses cannot be assigned to specific diagnostic structures. The manuscript therefore concludes that these visualisations should be interpreted only as qualitative representations of model behaviour rather than expert-validated localisation of diagnostic features (page 13, lines 345-352). The corresponding figure captions have also been revised to make clear that the displayed attention weights do not represent pathologist-validated regions of diagnostic importance.

5. The Discussion could further place the study within the broader methodological literature on robust and interpretable medical AI, digital biopsy, and independent multicenter validation. This is particularly relevant because the present study demonstrates excellent internal benchmark performance but appropriately recognizes that external validation is required before clinical translation. Related methodological studies may provide useful context, for example DOI: 10.1007/s00259-024-07024-x, DOI: 10.1007/s00259-025-07090-9, and DOI: 10.1186/s12916-025-03893-7. These studies could be discussed alongside other relevant literature to illustrate how explainability and independent multi-institutional validation are increasingly incorporated into clinically oriented AI frameworks.

Response: We thank the reviewer for this valuable suggestion. We have expanded the Discussion to place the present benchmark within the broader methodological development of robust and interpretable medical AI. In particular, we now discuss recent studies in which explainability, multi-institutional evaluation, and digital-biopsy concepts have been incorporated into clinically oriented imaging frameworks. These studies provide useful methodological context for the next stage of validation of EffAtt-LungNet, while addressing different diseases and imaging modalities from the present histopathology task.

The revised Discussion now emphasizes that strong internal benchmark performance alone is insufficient for clinical translation and that subsequent evaluation should incorporate independent multi-centre cohorts, variation across acquisition environments, and systematic expert assessment of model explanations. This discussion has been added on page 14, immediately following lines 390-394, where the manuscript already identifies independent multi-centre validation and pathologist-based assessment of attribution maps as necessary future directions.

Overall, the manuscript is technically interesting and potentially suitable for publication after minor revision, provided that the LC25000 source-image provenance and data-splitting procedure are clearly resolved.

Response: We thank the reviewer for the positive assessment of the manuscript and for considering the study potentially suitable for publication after minor revision.

Attachment Submitted filename: Response to Reviewers-effinet.docx

# Peer review: Decision Letter 1

**DOI:** 10.1371/journal.pone.0359302.r003

**Authors:** Chong Jiang

14 Sep 2026

EffAtt-LungNet: an attention-guided EfficientNet framework for three-class lung histopathology classification

PONE-D-26-38240R1

Dear Dr. Kajla,

We’re pleased to inform you that your manuscript has been judged scientifically suitable for publication and will be formally accepted for publication once it meets all outstanding technical requirements.

Within one week, you’ll receive an e-mail detailing the required amendments. When these have been addressed, you’ll receive a formal acceptance letter and your manuscript will be scheduled for publication.

An invoice will be generated when your article is formally accepted. Please note, if your institution has a publishing partnership with PLOS and your article meets the relevant criteria, all or part of your publication costs will be covered. Please make sure your user information is up-to-date by logging into Editorial Manager at Editorial Manager® and clicking the ‘Update My Information' link at the top of the page. For questions related to billing, please contact billing support.

If your institution or institutions have a press office, please notify them about your upcoming paper to help maximize its impact. If they’ll be preparing press materials, please inform our press team as soon as possible -- no later than 48 hours after receiving the formal acceptance. Your manuscript will remain under strict press embargo until 2 pm Eastern Time on the date of publication. For more information, please contact onepress@plos.org.

Kind regards,

Chong Jiang

Academic Editor

PLOS One

Additional Editor Comments (optional):

Dear Dr Kajla,

Thank you for submitting the revised version of your manuscript, “EffAtt-LungNet: an attention-guided EfficientNet framework for three-class lung histopathology classification.”

The manuscript has been substantially improved, and the reviewers’ main concerns have been satisfactorily addressed. The revised manuscript is now sufficiently clear, methodologically transparent, and appropriately interpreted.

I am therefore pleased to recommend acceptance of the manuscript for publication in PLOS ONE.

Please complete any remaining technical or data-availability requirements during the production process.

Sincerely,

Chong Jiang

Academic Editor

PLOS ONE

Reviewers' comments:

Reviewer's Responses to Questions

**Comments to the Author**

Reviewer #1: All comments have been addressed

Reviewer #2: All comments have been addressed

**********

2. Is the manuscript technically sound, and do the data support the conclusions?

Reviewer #1: Yes

Reviewer #2: Yes

**********

3. Has the statistical analysis been performed appropriately and rigorously?

Reviewer #1: Yes

Reviewer #2: Yes

**********

4. Have the authors made all data underlying the findings in their manuscript fully available?

The PLOS Data policy

Reviewer #1: Yes

Reviewer #2: Yes

**********

5. Is the manuscript presented in an intelligible fashion and written in standard English?

Reviewer #1: Yes

Reviewer #2: Yes

**********

Reviewer #1: (No Response)

Reviewer #2: (No Response)

**********

what does this mean?). If published, this will include your full peer review and any attached files.

If you choose “no”, your identity will remain anonymous but your review may still be made public.

**Do you want your identity to be public for this peer review?**  For information about this choice, including consent withdrawal, please see our Privacy Policy

Reviewer #1: No

Reviewer #2: No

**********

# Editor report: Acceptance letter

**DOI:** 10.1371/journal.pone.0359302.r004

**Authors:** Chong Jiang

PONE-D-26-38240R1

PLOS One

Dear Dr. Kajla,

I'm pleased to inform you that your manuscript has been deemed suitable for publication in PLOS One. Congratulations! Your manuscript is now being handed over to our production team.

At this stage, our production department will prepare your paper for publication. This includes ensuring the following:

* All references, tables, and figures are properly cited

* All relevant supporting information is included in the manuscript submission,

* There are no issues that prevent the paper from being properly typeset

You will receive further instructions from the production team, including instructions on how to review your proof when it is ready. Please keep in mind that we are working through a large volume of accepted articles, so please give us a few days to review your paper and let you know the next and final steps.

Lastly, if your institution or institutions have a press office, please let them know about your upcoming paper now to help maximize its impact. If they'll be preparing press materials, please inform our press team within the next 48 hours. Your manuscript will remain under strict press embargo until 2 pm Eastern Time on the date of publication. For more information, please contact onepress@plos.org.

You will receive an invoice from PLOS for your publication fee after your manuscript has reached the completed accept phase. If you receive an email requesting payment before acceptance or for any other service, this may be a phishing scheme. Learn how to identify phishing emails and protect your accounts at https://explore.plos.org/phishing.

If we can help with anything else, please email us at customercare@plos.org.

Thank you for submitting your work to PLOS One and supporting open access.

Kind regards,

PLOS One Editorial Office Staff

on behalf of

Dr. Chong Jiang

Academic Editor

PLOS One
